# The transcriptome of rabbit conjunctiva in dry eye disease: Large-scale changes and similarity to the human dry eye

**DOI:** 10.1371/journal.pone.0254036

**Published:** 2021-07-29

**Authors:** Adam Master, Apostolos Kontzias, Liqun Huang, Wei Huang, Anna Tsioulias, Samaneh Zarabi, Michael Wolek, Brian M. Wollocko, Robert Honkanen, Basil Rigas

**Affiliations:** 1 Department of Preventive Medicine, Stony Brook University, Stony Brook, New York, United States of America; 2 Department of Medicine, Stony Brook University, Stony Brook, New York, United States of America; 3 Medicon Pharmaceuticals, Inc., Setauket, New York, United States of America; 4 Department of Ophthalmology, Stony Brook University, Stony Brook, New York, United States of America; 5 Department of Pathology, Stony Brook University, Stony Brook, New York, United States of America; 6 Renaissance Medical School, Stony Brook University, Stony Brook, New York, United States of America; Rutgers Biomedical and Health Sciences, UNITED STATES

## Abstract

The pathophysiology of dry eye disease (DED) remains largely unknown, accounting in part for the lack of successful treatments. We explored the pathophysiology of DED using a rabbit model of chronic DED induced with 3 weekly injections of Concanavalin A into the periorbital lacrimal glands. The transcriptome of full-thickness’s conjunctival tissue from rabbits with DED and from normal controls was determined using microarrays and, as needed, confirmatory real-time polymerase chain reactions. Results were subjected to bioinformatic analysis. DED induced large-scale changes in gene transcription involving 5,184 genes (22% of the total). Differentially expressed genes could be segregated into: functional modules and clusters; altered pathways; functionally linked genes; and groups of individual genes of known or suspected pathophysiological relevance to DED. A common feature of these subgroups is the breadth and magnitude of the changes that encompass ocular immunology and essentially all aspects of cell biology. Prominent changes concerned innate and adaptive immune responses; ocular surface inflammation; at least 25 significantly altered signaling pathways; a large number of chemokines; cell cycle; and apoptosis. Comparison of our findings to the limited extant transcriptomic data from DED patients associated with either Sjogren’s syndrome or non-Sjogren’s etiologies revealed a significant correlation between human and rabbit DED transcriptomes. Our data, establishing the large-scale transcriptomic changes of DED and their potential similarity to the human, underscore the enormous complexity of DED; establish a robust animal model of DED; will help expand our understanding of its pathophysiology; and could guide the development of successful therapeutic strategies.

## Introduction

Dry eye disease (DED) is a multifactorial disease characterized by disrupted tear film homeostasis on the ocular surface and accompanied by ocular symptoms triggered by hyperosmolarity, ocular surface inflammation and neurosensory abnormalities [1–3]. DED affects 1 in 6 humans with an estimated prevalence of 15%, and has a staggering impact on the economy [4]. DED is classified into two major groups: aqueous deficient (decreased production of the aqueous component of the tear film; ~20% of DED) and evaporative (increased evaporation of the tear film; ~50% of DED). About 30% of DED patients show evidence of both (mixed DED) [5]. DED symptoms range widely from mild discomfort to the devastating loss of vision or neuropathic ocular pain requiring narcotics for its control [6]. Current therapies of DED, including topically administered pharmacological agents, are considered suboptimal. A thorough understanding of the pathogenesis of DED is deemed crucial if further progress in its treatment is to be made.

Under physiological conditions, a tight regulation maintains the homeostasis of the tear including its optical properties. In DED, perturbation of homeostasis leads to tear film instability and visual disturbances, thus contributing to ocular surface damage [3]. Several etiologies of DED promote an unstable and hyperosmolar tear film leading to inflammation, its core mechanism [7,8]. Remarkably, oxidative stress, the result of environmental factors, acute and chronic diseases and even normal aging contributes to the pathogenesis of DED, and it may prove a crucial early event [9,10]. The pathogenesis of DED has been conceptualized as “the vicious cycle of DED.” Key participants in this vicious cycle are ocular surface desiccation; tear hyperosmolarity; ocular surface inflammation; and barrier disruption by lysis of epithelial tight junctions, accelerating apoptotic cell death. Ample evidence has substantiated this vicious cycle [8]. Stress signaling triggered by desiccation and hyperosmolarity leads to the nonspecific innate immune response, followed by the slower, but more specific adaptive immune response, in which CD4^+^ T cells damage the ocular surface. In particular, such signaling events initiate an acute inflammatory response, which promotes the production of proinflammatory cytokines (IL-1α, IL-1β, IL-6, TNF-α), chemokines (CCL3, CCL4) and the apparently important metalloproteinases (e.g., MMP-9) mediated by MAPK and NF-κB signaling [11]. Activation and recruitment of inflammatory cells to the ocular surface ensues, propagating the production of inflammatory mediators. Ultimately, the expression of mucins is reduced, and ocular surface epithelial cells undergo apoptotic death including loss of goblet cells [12].

During the innate immune response, natural killer cells, dendritic cells, macrophages and CD4^+^ CD8^+^ T cells, when activated, respond readily to stimuli generated by stress signaling in DED [13]. The elements of the vicious cycle are evident here as well. For example, proinflammatory cytokines stimulate the maturation of antigen presenting cells, which in turn facilitate T cell differentiation (memory T cells, T_H_1, T_H_2, T_H_17, T_reg_), which are recruited to the ocular surface. In the absence of appropriate constraints, the immune response is amplified by dysregulated effector T cells that enhance inflammation and damage, followed by more proinflammatory cytokines. Epithelial changes, part of this pathogenetic spiral, further destabilize the tear film, amplify inflammation, and propagate the vicious cycle of DED. Indeed, as aptly stated, to control DED one must “break the cycle” [9].

Animal models of DED have been used extensively to elucidate its pathophysiology and in preclinical drug development [10,11]. The rabbit offers distinct advantages as an animal model of DED. Rabbits can be easily handled, while sharing more common anatomical and biochemical features with humans compared to rodents, including longer life span and larger eye size [11]. We have recently developed and validated two rabbit models of DED suitable for a broad assessment of its pathophysiology and evaluation of therapeutic agents [14–17]. One is based on the induction of dacryoadenitis by injecting the mitogen Concanavalin A (ConA) into the orbital lacrimal glands and the other on complete surgical dacryoadenectomy.

The present study, using the ConA model of chronic DED, interrogated conjunctival gene expression to identify critical pathways of the disease. Our findings demonstrating extensive differential gene expression in DED reveal its great complexity, reflected in changes in multiple signaling pathways. We also show a significant correlation between our results and those from patients with DED, suggesting that this rabbit model is a valid experimental tool for the study of DED pathogenesis and for the preclinical evaluation of candidate therapeutics.

## Materials and methods

### Rabbits

Male Dutch-Belted rabbits, 1.8–2 kg (Covance Research Products, Somerset, NJ) were housed singly under strict temperature (70 ± 5 °F) and humidity (45± 5%) control and acclimated for at least 2 weeks before the induction of DED. They had unlimited access to water and standard rabbit chow without dietary enrichment. The nictitating membranes were removed surgically during the early acclimation period.

All animal studies were approved by the Institutional Review Board of Stony Brook University (ID# 714306) and performed in accordance with the Association for Research in Vision and Ophthalmology Statement for the Use of Animals in Ophthalmic and Vision Research. Rabbits were euthanized with an IV injection via an ear vein of Fatal-Plus (pentobarbital sodium 390 mg/ml) Solution from Vortech Pharmaceuticals, Dearborn, MI, USA.

### Induction of chronic DED

As described in detail elsewhere [11], the periorbital lacrimal glands of rabbits were injected with ConA on days 1, 8 and 14 to induce DED. Tear osmolarity, tear break-up time, tear production and corneal sensitivity were measured at baseline and at the conclusion of the study on day 18 when the animals were euthanized. Tears were collected (at the junction of the palpebral and bulbar conjunctivas along the lower fornix) and tear osmolarity was measured with the same instrument, a TearLab Osmometer (TearLab Corporation, San Diego, CA). *Tear break-up time* (TBUT) was determined after application of a drop of 0.2% fluorescein over the ocular surface. The pre-corneal tear film was observed and the time until the development of black dots, lines or obvious disruption of the fluorescein film was measured up to a maximum of 60 sec. Tear production was determined using the *Schirmer tear test* (STT). The STT was performed in triplicate by inserting the appropriate strips (EagleVision, Denville, NJ) at the midpoint of the lower lid. The average of the length of the moistened strip at 5 min was recorded. *Corneal sensitivity (CS)* was measured with the Cochet-Bonnet aesthesiometer (Luneau, France) [18]. The aesthesiometer contains a thin nylon filament that is applied perpendicularly to the central cornea, initially at its full length of 6 cm. If a positive response (full blink and retraction of the eye into the ocular orbit) is not noted on 3–5 attempts, the filament is incrementally retracted in 5 mm steps until a clear stimulus is evoked. The length of filament simulating a positive response was recorded.

For transcriptome studies, 12 animals were randomly divided into two experimental groups: Naïve and rabbits with ConA-induced DED. Due to the small size of the conjunctival tissues, RNA from 2 animals (4 eyes) was pooled for each microarray (three per group). The sample size was based on our previous extensive experience with this animal model [11].

### RNA isolation

After each rabbit was euthanized, a 4x4 mm sample of full-thickness conjunctiva tissue that included Tenon’s capsule was immediately placed in 1 mL of TRIzol Reagent (ThermoFisher Scientific, CA), frozen in liquid nitrogen and stored at -80 °C until analyzed. Samples were homogenized in TRIzol, mixed with 0.2 mL of chloroform, vortexed 20 s and centrifuged at 12,000 *× g* at 4°C for 10 min. Pre-cleaned RNA, present in the upper aqueous phase, was transferred to a new tube on ice and processed using the RNeasy^®^ Mini column kit (Qiagen, Valencia, CA) with DNase digestion during RNA purification.

### Gene expression microarrays

RNA quality was evaluated with a 2100 Bioanalyzer (Agilent Technologies) that assessed the relative abundance of the 18S and 28S ribosomal bands and the presence of baseline rise. 150 ng of total RNA was labeled for hybridization to Affymetrix GeneChip^™^ Rabbit Gene 1.0 ST Array (ThermoFisher Scientific) containing 496,321 probes to assess 23,364 transcripts, transcript variants and alternative splicing events. Microarray data sets were deposited in NCBI Gene Expression Omnibus (GEO) under the Accession Number GSE171043. The total RNA was labeled using the Affymetrix GeneChip^®^ WT PLUS Kit according to the manufacturer’s protocol. Once the hybridization was completed, the arrays were washed and stained in a GeneChip Fluidics 450 station. These arrays were then scanned in a GeneChip Scanner 3000 G7 controlled by Affymetrix GeneChip Command Console v 4.3.3.1616. Each transcript was measured with a median of 22 probes. Of these 23,364 transcripts, 14,713 (63%) are fully annotated, with known homology to human genes (HomoGene NCBI database) while 8,651 (37%) are partially annotated without clear homology to human genes.

### Reverse transcription and RT-PCR

RNA concentration and purity were assessed using a NanoDrop 2000C Spectrophotometer (ThermoFisher Scientific). For cDNA synthesis and genomic DNA elimination in RNA samples RT2 Profiler PCR Arrays was used (Qiagen, Valencia, CA). Real Time PCR (RT-PCR) was performed with RT2 SYBR Green Fluor qPCR Mastermix (Qiagen, Valencia, CA) used with predesigned array plates on MyiQ^™^ Real-Time PCR Detection System (Bio-Rad). These plates, RT^2^ Profiler^™^ PCR Array Rabbit Innate & Adaptive Immune Responses PANZ-052ZA 96-Well Format (Qiagen, Valencia, CA), detect the expression of 84 tested genes, 5 housekeeping genes and other controls. The PCR protocol consisted of one cycle at 95°C (10 minutes) followed by 40 cycles of 95°C (15 sec) and 60°C (1 min) followed by fluorescence data collection (SYBR Green channel) confirmed then by melting curve analysis. Fold changes in gene expression were determined using the C_*t*_ method with normalization to ACTA2, ACTB, GAPDH, LDHA, LOC100346936 controls. To calculate mRNA levels, we used the comparative C_*t*_ method (^ΔΔ^C_*t*_ method) [19].

### Bioinformatic analyses

Microarray results were analyzed with the Transcriptome Analysis Console (TAC) Software (ThermoFisher Scientific). Real-Time PCR results were analyzed with an excel-based software -RT2 Profiler PCR Array provided by Qiagen with installed PANZ-052ZA PCR Array, Rt^2^ Profiler PCR Array Analysis software, Version 3.5^™^; and QuantStudio^™^ 12 K Flex software (ThermoFisher Scientific, Waltham, MA). The expression of all genes of interest is presented as fold change relative to naive rabbits. Further pathway-focused gene analyses were performed based on the Kyoto Encyclopedia of Genes and Genomes (Kegg) Pathway online database [20] and the “Enrichr” gene enrichment analysis online tool [21] and Reactome online database [22].

Clustering analysis of the microarray results was performed with String software (https://string-db.org, organism: Oryctolagus cuniculus, gene network clustered to MCL inflation parameter: 1.9).

The human microarray data of conjunctival epithelium of patients with severe DED, deposited as GSE28941 DataSets [23] in NCBI’s Gene Expression Omnibus (GEO), were analyzed with GEO2R software provided by NCBI GEO and correlated with our rabbit microarray data.

Human gene expression data obtained with NanoString^®^ nCounter technology by Liang *et al* [24] were correlated with our rabbit microarray data.

### Statistical analyses

For statistical analysis of microarray data, we used statistical protocol included in Transcriptome Analysis Console (TAC) Software, which used the Robust Multi-chip Analysis (RMA) algorithm as multi-chip analysis approach. Fold change analysis was used to compare the mean expression levels of various transcripts in the normal conjunctival tissue compared to the con-A treated conjunctival tissues. The genes showing a 1.5-fold change (increase or decrease) relative to normal conjunctival tissue were identified and tabulated, with their corresponding p values.

Differences among groups were analyzed by one-way ANOVA [23] with Newman-Keuls post-tests, while correlations were established according to the Pearson’s product moment correlation coefficient [25].

PCR-array results are presented as *Mean ± SD* and analyzed by Student’s t-test using the 12 K Flex QuantStudio^™^ software (ThermoFisher Scientific, Waltham, MA). Differences among groups and correlations among different parameters were considered statistically significant when p < 0.05.

## Results

### The induction of DED in rabbits

As expected [11], the three weekly injections of ConA, established chronic DED in the rabbits. Compared to baseline, on day 18 the naive group showed limited, statistically not significant changes in all four parameters of DED that we evaluated (Table 1). In contrast, rabbit injected with ConA developed full-blown DED evidenced by marked and significant changes in these parameters, all in the direction of DED. Specifically, tear osmolality increased, while TBUT, STT and corneal sensitivity, all decreased. by 73.5% 38%; all changes were statistically significant (p = 3.76e 07 to 0.002) Control rabbits showed no such changes.

**Table 1:** ConA-induced dry eye disease in rabbits.

	Baseline	Day 18	% change; P value
	*Mean ± SEM*		
**Osmolality**, *mOsm/kgH*_*2*_*O*			
naive	286.8±3.8	281.7±2.3	-1.8%; NS
DED	283.1±.3.4	306.4±4.9	+8.2%; P<0.001
**TBUT**, *sec*			
naive	36.7±9.3	40.5±4.0	+10.3%; NS
DED	35.1±6.5	9.3±1.4	-73.5%; P<0.0005
**STT**, *mm (at 5 min)*			
naive	14.5±1.1	14.0±1.6	-3.4%; NS
DED	16.9±2.0	2.4±0.9	-85.8%; P<0.03
**CST**, *filament length*, *cm*			
naive	5.1±0.4	5.3±0.2	+3.9%; NS
DED	5.5±0.0	3.4±0.2	-35.8%; P<0.0001

NS, not statistically significant. *n* = 6–8 eyes/group.

Evaluation of these DED parameters using principal component analysis (PCA) [15] confirmed the induction of DED in the ConA-treated rabbits (mean PCA score = -0.72; p = 6.08x10^-10^; mean PCA score at baseline = 1.86).

**Fig 1:**
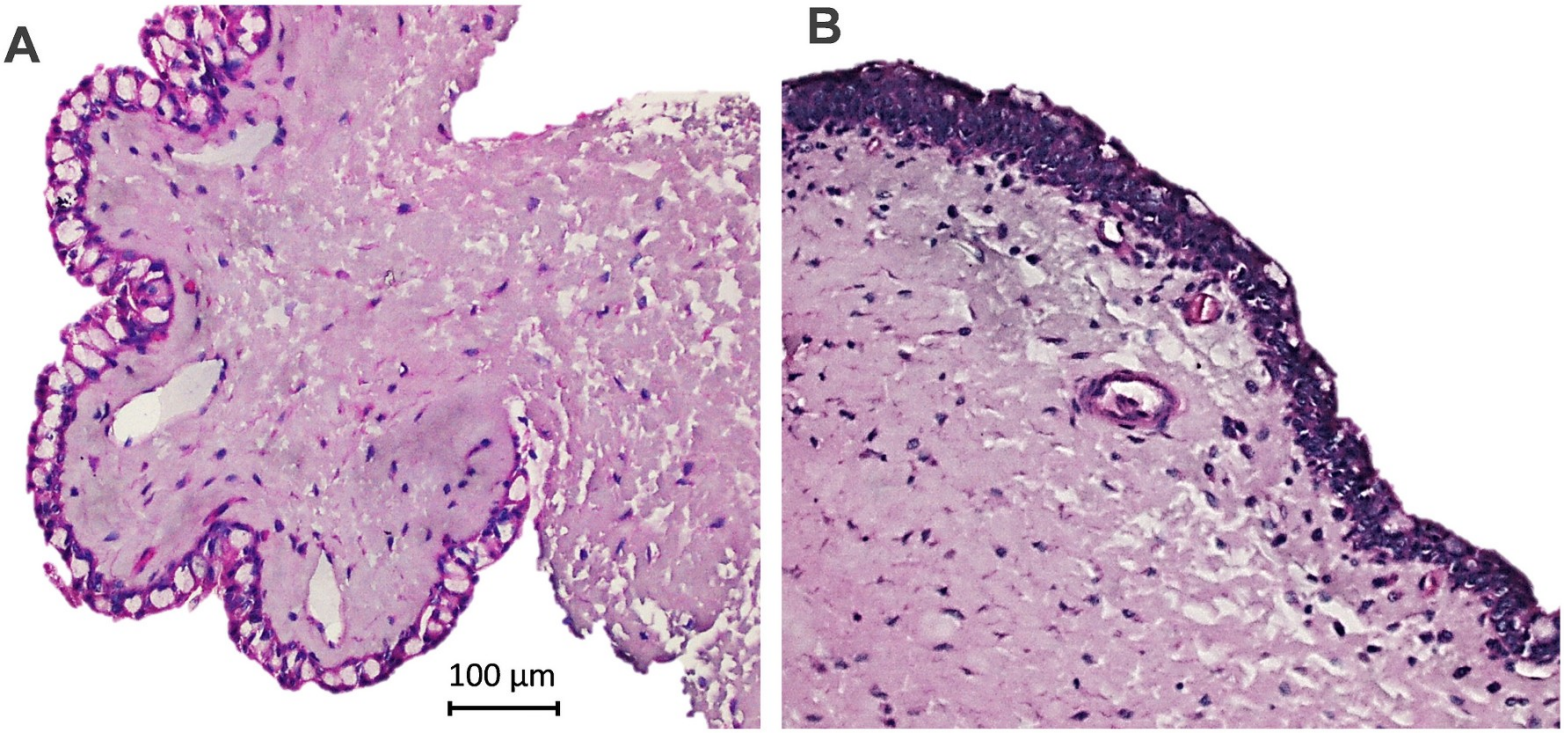
DED histopathology of the rabbit. Cross tissue sections of superior bulbar conjunctiva on day 18 from NZW rabbits (H&E stain). Representative images from naïve (A) and rabbis with DED (B), with the latter showing disrupted architecture (increased stratification, goblet cell loss, and chronic inflammatory infiltrate) not present in the normal tissue. Scale bar = 100 μm.

Histopathological evaluation of the conjunctiva of NZW rabbits with and without DED harvested on day 18 was performed. As expected, normal rabbits showed normal architecture with stratified nonkeratinized squamous epithelium with numerous goblet cells covering a vascular lamina propria. In contrast, ConA-treated animals with DED showed disrupted architecture, characterized by increased epithelial stratification, loss of goblet cells and higher density of inflammatory cells in the substantia propria (Fig 1) [14].

### Extensive differential gene expression in the conjunctiva of rabbits with DED

**Fig 2:**
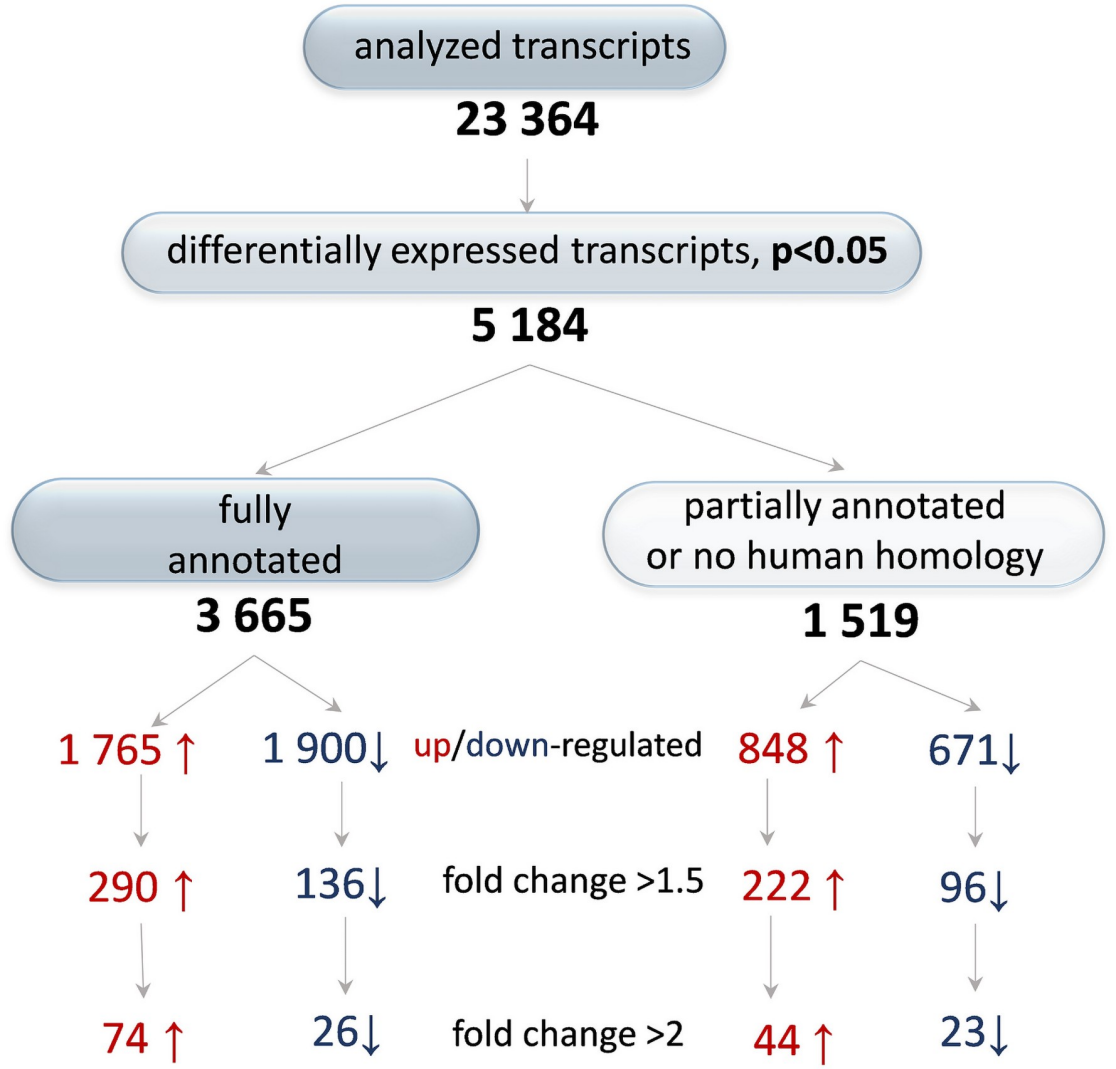
Differential gene expression in the conjunctiva of rabbits with DED. Transcriptome profiling of conjunctival tissue from rabbits with DED and normal controls was determined using microarrays as in Methods. The numbers of upregulated (red arrows) and downregulated (blue arrows) genes are shown; they are based on their annotation or human homology.

Transcript analysis of impression cytology samples from DED patients using high-throughput methods such as PCR, qPCR, RNA-Seq or microarrays has been reported [23,26,27]. Although informative, these studies are limited by analyzing a narrow segment of the transcriptome and by partial sampling of the conjunctiva. Impression cytology samples the uppermost cells of the ocular surface, whereas DED involves its entire thickness. Thus, it is likely that studies of impression cytology samples may overlook pathways critical to DED. Consequently, we studied the entire transcriptome of the conjunctiva and used RNA extracted from full-thickness samples of normal and DED rabbits. As shown in Fig 2, we analyzed 23,364 transcripts [27,28] anf those, 5,184 were differentially expressed between the two groups, representing slightly over 22% of the total. Most of the differentially expressed genes were fully annotated, while less than a third are partially annotated or lack human homology. Using the fold change (FC) value of >1.5, nearly half of the differentially expressed genes were upregulated in DED and the other half (FC <-1.5) were downregulated. With FC ≥2, only 100 fully annotated genes (2.7% of the total) and 67 of the partially annotated genes (1.3%) showed such (marked) change in their expression.

#### Cluster analysis

The clustering of gene expression data is a very useful approach to identify the natural structure inherent in gene expression data, and to understand gene functions, cellular processes, and subtypes of cells, and, importantly, gene regulation [29]. Thus, we applied cluster analysis to the initial evaluation of our results.

**Fig 3:**
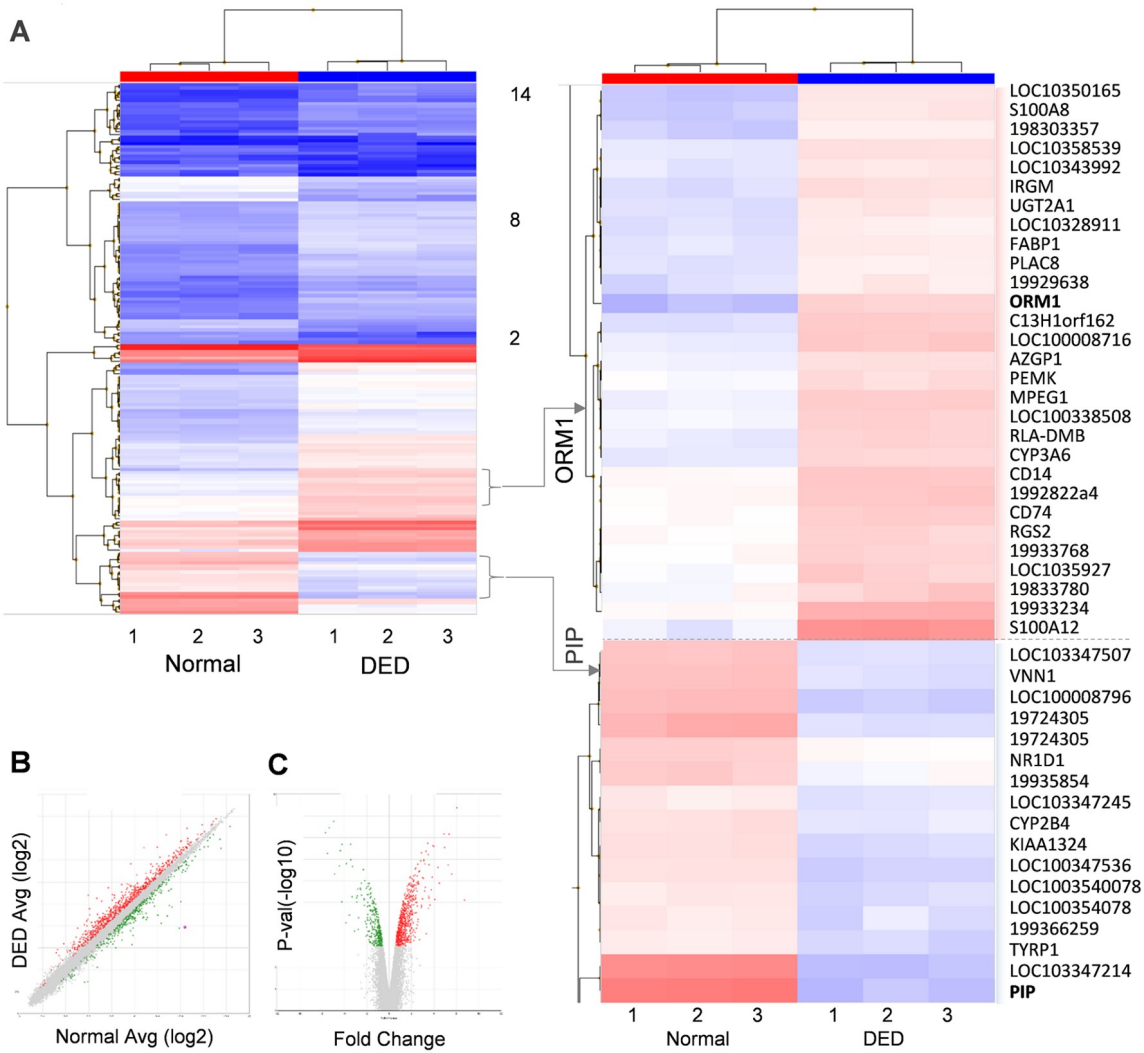
Cluster analysis of gene expression in normal and DED rabbits. **A.** Heatmap of 831 differentially expressed gene transcripts from Control (left) and DED (right) rabbits (n = 3 each). Red and blue colors denote upregulation and downregulation, respectively. **B:** Scatterplot representing the data distribution between DED and normal rabbits. **C:** The volcano plot depicts the statistical significance (p value) versus fold change in geneexpression. Two subclusters of the genes overex-pressed (ORM1) or downregulated (PIP) in DED rabbits compared to normal contols (enlargement to right of A).

We evaluated the level of expression of genes in normal and DED rabbits (three samples from each). Hierarchical clustering based on FC>1.25 identified 831 gene transcripts that were differentially expressed between DED and normal rabbits (p<0.001). Significant differences between normal and DED animals are evident in the cluster heat map of Fig 3. Among the clusters with the statistically strongest significant differences are the orosomucoid 1 (ORM1) and the prolactin-inducible protein (PIP) clusters. There are 29 upregulated genes in the ORM1 cluster and 16 downregulated genes in the PIP cluster. Interestingly, proteomic analyses of tear fluid identified ORM1 and PIP as biomarkers of DED in humans [30,31]. The statistical significance (p value) versus magnitude of change (fold change) in these results is visualized in the two scatter plots of Fig 3 (the latter being a volcano plot).

**Fig 4:**
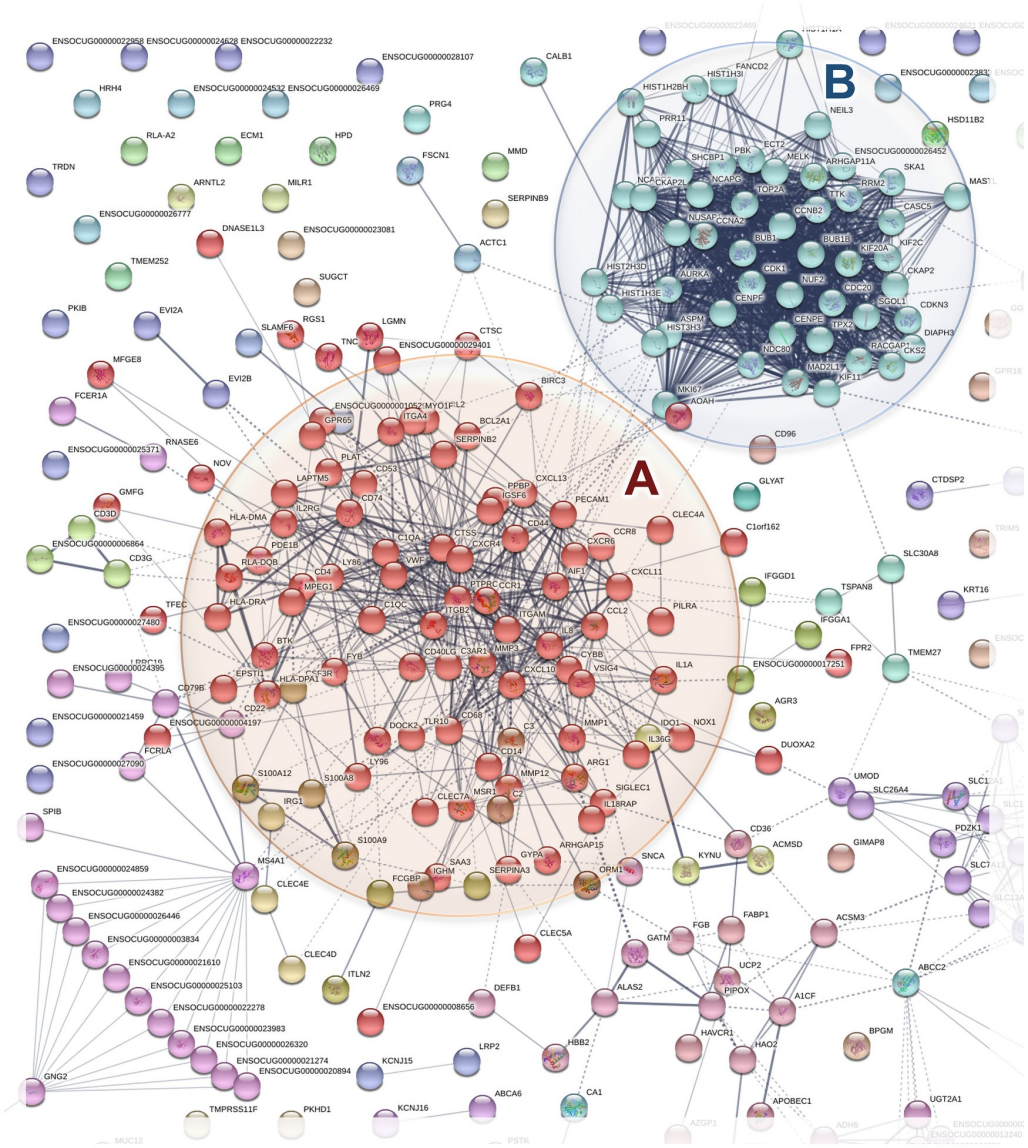
Clusters of immune response and cell cycle regulation in DED. Of the genes upregulated (FC >1.5, p<0.05) in rabbit DED, 55% were in two clusters, **A** (red balls), corresponding to the innate and adaptive immune response, and **B**, corresponding to the cell cycle regulating gene network (blue balls). The analysis was performed as in Methods.

*Protein–protein associations*. Protein–protein associations are a useful concept to group all protein-coding genes in a genome [31,32]. The complete set of associations can be assembled into a large network, which captures the current knowledge on the functional modularity and interconnectivity in the cell. The space of potential protein–protein interactions is much larger, and also more context-dependent, than the space of intrinsic molecular function of individual molecules. We used the STRING database which is designed to comprehensively assemble and evaluate protein–protein association information [33]. Among the changes in gene expression in DED compared to normal, we identified the two prominent functional networks of known and predicted protein–protein interactions shown in Fig 4. They correspond to upregulated genes (FC >1.5, p<0.05) of innate and adaptive immune response (cluster A) and cell cycle regulation (cluster B) [33]. The gene network clustered to MCL; inflation parameter: 1.9. Of interest, no clearly identifiable clusters were observed in downregulated genes (S1 Fig).

*Selected gene transcripts*. Of the differentially expressed fully annotated rabbit genes, 426 were either up- or down-regulated based on an absolute cut-off value of 1.5 (S1 Table).

Table 2 presents a selection of genes from the various subgroups of these genes. This selection was based on an arbitrary cut-off point for each subgroup and the perceived importance of the selected genes to each pathway. The contribution of these genes to DED is uncertain, given the limitations of our understanding of its pathophysiology and the inferential nature of their analysis. Nevertheless, some of these genes are plausible participants in DED pathogenesis, as discussed below.

**Table 2:** Selected differentially expressed genes based on fold change.

	Gene	Fold change	p-value
**Innate immunity**			
Neutrophil degranulation	S100A12	8.49	2.90E-14
	CHI3L1	3.89	3.20E-10
	S100A8	3.72	1.00E-10
	CXCR1	2.67	4.7 e-9
	CYBB	2.31	4.9 e-8
	CXCR2	2.28	5.0 e-8
	ITGAM	2.25	5.5 e-8
**Antigen presentation and adaptive immunity**			
MHC class II antigen presentation	HLA-DQA1	3.17	9.20E-12
	HLA-DRB1	2.38	6.50E-04
	HLA-DRA	2.29	1.90E-10
	HLA-DMA	1.88	2.30E-07
TCR and PD-1 signaling	CD3D	1.91	2.0 e-6
	FYB	1.86	5.8 e-5
	CD3G	1.74	2.0 e-6
	CD4	1.55	0.0005
**Cytokine signaling**			
Interleukin 10 signaling	CXCL10	2.07	2.4 e-7
	FPR2	1.99	0.002
	IL1A	1.91	1.0 e-4
	CCR1	1.79	1.6 e-5
	ALDH2	-1.7	2.2 e-6
	Mar-01	-1.67	4.5 e-8
	PADI3	-1.67	1.6 e-6
	LY6G6C	-1.63	6.8 e-8
	LSAMP	-1.63	3.1 e-7
	PLCB4	-1.63	1.0 e-6
	DOK5	-1.63	1.2 e-5
	RAMP2	-1.63	0.001
Interleukin 12 signaling	CA1	2.77	1.0 e-7
Interleukin 1 signaling	IL18RAP	1.51	0.013
	IL36G	1.51	0.01
	CASP1	1.21	2.0 e-4
Interleukin 4 and 13 signaling	LCN2	-2.03	1.0 e-6
**Signal transduction**			
Chemokine receptors bind chemokines	CXCL6	3.54	8.7 e-11
	CXCL11	3.07	7.0 e-8
	CXCL8	2.38	1.5 e-8
	CCL7	2.37	4.5 e-8
	CXCR4	2.21	1.1 e-6
	CXCL10	2.07	2.4 e-7
	CXCL13	1.92	1.0 e-6
	CCR1	1.79	1.6 e-5
	CCRL2	1.72	1.2 e-7
	CXCR6	1.54	8.5 e-5
Signaling by NOTCH4	HEY1	-1.63	4.0 e-6
	HEY2	-1.62	5.5 e-5
Signaling by Rho-GTPase	NOX1	2.3	6.3 e-9
	RACGAP1	1.61	1.7 e-5
Signaling by GPCR	GPR18	2.25	3.0 e-6
	HTR1B	-2.36	4.0 e-7
	OPN5	-1.91	1.9 e-7
	CCL21	-1.87	7.7 e-9
Signaling by BCR	CD79B	1.6	4.0 e-6
Signaling by Wnt	WIF1	-2	3.6 e-8
**Extracellular matrix organization**			
Integrin cell surface interactions	MMP12	3.06	3.7 e-8
	MMP3	1.56	1.0 e-6
	MMP1	1.51	4.3 e-5
	MMP13	1.29	0.002
	MMP9	1.17	0.039
Degradation of extracellular matrix	ADAMTS1	-1.58	1.0 e-6
	CAPN9	-1.51	1.0 e-6
**Apoptosis**			
Caspases	CASP3	1.44	5.9 e-5
	CASP10	1.37	7.0 e-4
	CASP1	1.21	2.0 e-4
**Metabolism**			
Transport of small molecules	SLC13A1	2.3	2.2 e-9
	SLC5A12	2	4.4 e-9
	SLC17A1	2	1.0 e-6
	SLC3A1	1.94	4.3 e-8
	SLC16A9	1.92	2.1 e-7
	SLC36A2	1.89	1.0 e-5
	SCGB1D1	1.68	1.0 e-6
	SLC7A3	-2.17	9.0 e-6
Oxidation and oxidative stress mediated transcription	NOX1	2.3	6.3 e-9
	GSTA2	2.26	1.2 e-8
	PIPOX	1.63	4.0 e-4
	HAO2	1.6	1.0 e-6
	GPX3	1.54	0.0003
	AOX4	-1.75	9.0 e-6
	ABCA6	-2.77	1.43 e-7
	FBXO32	-1.58	0.001

Notable among the upregulated genes are the following. *S100A8* (FC 3.72, p = 1.0e-10) corroborates prior evidence that S100 proteins induce inflammation by activation of mitogen-activated protein kinase (MAPK)/nuclear factor kappa (NF-κB) pathways, which favor inflammatory changes in DED. *S100A8* was elevated in a proteomic biomarker study of DED patients compared to healthy subjects [33]. It has been postulated that *S100A8* is pivotal in DED pathogenesis by inducing apoptosis [34] as observed in our microarrays by upregulation of *CASP3* (FC 1.44, p = 5.9e-5) and *CASP10* (FC 1.37, p = 2.0e-4). *S100A8* production by macrophages requires the presence of reactive oxygen species (ROS) [34]. Our data depict upregulation of genes involved in oxidation pathways such as *NOX1* (FC 2.30, p = 6.3e-9) and *GSTA2* (FC 2.26, p = 1.2e-8). We also observed upregulation of *HLA-DRB1* (FC 2.38, p = 6.5e-4) and *HLA-DRA* (FC 2.29, p = 1.9e-10), in line with reports of an association of increased expression of major histocompatibility complex (MHC) II molecules with DED [24].

Among the downregulated genes of importance are *HEY1* (FC -1.63, p = 4.0e-6), *HEY2* (FC -1.62, p = 5.5e-5) and *WIF1* (FC -2.0, p = 3.6e-8), which are involved in the NOTCH and WNT pathways, both downregulated in human DED [23]. It is hypothesized that NOTCH and WNT signaling plays a role in the pathogenesis of DED by altering the development of non-goblet and goblet cells.

### Reactome analysis

Reactome databases afford the opportunity to explore molecular details of several cellular processes [22,35]. Such exploration is possible because the Reactome database, which generalizes the concept of a reaction, includes nucleic acids, small molecules, proteins, and macromolecular complexes. Various biological processes including signaling, metabolism, transcriptional regulation, transport, and others can be analyzed in a single format. Thus, we employed reactome-based analysis of genes with significantly changed expression in DED compared to normal. Of the 3,665 fully annotated differentially expressed genes, 2,318 were found in Reactome.

#### Functional modules

This analysis revealed that 55% of upregulated genes in DED (FC >1.5, p<0.05) were segregated in discrete functional modules including genes involved in: a) innate and adaptive immune response, e.g., chemokines, GPCRs, TLRs, MHC class II antigen presentation (87 genes, 23.3%), b) cell cycle checkpoints, mitotic metaphase and anaphase, kinetochores (51 genes, 13.8%), c) SLC (solute carrier) -mediated transmembrane transport, ABC transporters (20 genes, 5.4%), d) G proteins, signaling through PI3Kγ, PLCβ, prostacyclin, potassium channels, aquaporins (12 genes, 3.3%), e) metabolism of drugs such as oxidation, glucuronidation, phase I and II—functionalization of compounds, PPARA, regulation of lipid metabolism (12 genes, 3.3%), f) translational control, ribosome processing, selenocysteine synthesis, SRP targeting, nonsense mediated decay (NMD), rRNA processing, mRNA editing (7 genes, 1.9%), and g) β defensins, antimicrobial peptides, metabolism of amino acids and derivatives (6 genes, 1.6%) as well as other smaller gene clusters found to be upregulated.

Of the genes downregulated in DED (FC <-1.3, p<0.05), 16% were found in functional modules. These genes include genes involved in: a) transcriptional control, nuclear receptors, cellular responses to stress, detoxification of ROS, A/1 rhodopsin-R (15 genes, 3.3%), b) 40S ribosome turnover, selenocysteine synthesis, Nonsense Mediated Decay (NMD) (13 genes, 2.9%), c) metabolism, oxidation, TNF signaling, death receptor signaling, sulfide turnover (12 genes, 2.7%), d) lipid, sphingolipid and inositol metabolism, cell differentiation, triglyceride catabolism (8 genes, 1.8%), e) WNT/TCF signaling, estrogen signaling, intrinsic apoptosis, BCL-2 regulation, IL-4 and IL-13, YAP1-mediated transcription, (8 genes, 1.8%), cytoskeleton turnover, signal transduction, axon guidance, Ca-dependent regulation, FOXO-mediated transcription, transport to the Golgi (7 genes, 1.6%), as well as other smaller gene clusters that were downregulated (S1 Table and S1 Dataset).

*Pathway analysis*. We also examined whether transcripts with altered expression associated with DED may affect specific pathways; such changes would be more likely to be relevant to DED pathogenesis. This analysis is a statistical (hypergeometric distribution) test that determines whether certain pathways are enriched in the microarray data. The probability score for each candidate pathway was obtained and corrected for false discovery rate (FDR) using the Benjamani-Hochberg statistical method. All non-human identifiers were converted to their human equivalent.

Of the pathway inferences generated by our analysis, the 25 statistically most significant pathways are listed in Table 3. It is interesting that all these pathways participate in pivotal immune and metabolic events [5,10]. We have grouped them according to the mechanisms involved in the pathogenesis of DED. Notable among them are pathways mediating the innate and adaptive immune responses, both early events in DED; gene transcription; cell signaling; and translation (S1 Fig and S1 Dataset).

**Table 3:** The 25 most involved pathways in rabbit DED.

Pathway	p-value
***Innate immune response***	
Neutrophil degranulation	8.81e-04
Chemokine receptors bind chemokines	0.005
MHC class II antigen presentation	0.006
Interleukin-10 signaling	0.002
***Adaptive immune response***	
Phosphorylation of CD3 and TCR zeta chains	0.002
TCR signaling	0.009
Downstream TCR signaling	0.011
PD-1 signaling	0.008
Translocation of ZAP-70 to Immunological synapse	0.004
***Gene transcription***	
TFAP2 family regulates transcription of growth factors and their receptors	0.01
Transcriptional regulation by RUNX3	0.011
Nuclear Receptor transcription pathway	0.009
Transcriptional regulation by the AP-2 family of transcription factors	0.007
Nonsense Mediated Decay independent of the Exon Junction Complex	0.004
Regulation of expression of SLITs and ROBOs	0.003
Generation of second messenger molecules	0.014
Prefoldin mediated transfer of substrate to CCT/TriC	0.014
Immune cell trafficking and signaling	
Signaling by ROBO receptors	0.007
Polo-like kinase mediated events	0.009
Translation and post-translational modifications	
L13a-mediated translational silencing of ceruloplasmin expression	0.009
GTP hydrolysis and joining of the 60S ribosomal subunit	0.006
Formation of a pool of free 40S subunits	0.006
Peptide chain elongation	0.004
Eukaryotic Translation Elongation	0.005
Eukaryotic Translation Termination	0.015

### Gene expression changes of potential pathophysiological relevance

To further assess the potential pathophysiological role of the gene expression changes that we observed, we performed a structured literature search, which examined whether the differentially expressed genes identified by our microarray analysis have been previously linked to DED. Terms used in this PubMed and Google Scholar search for publications from 2009 to 2019 included “dry eye syndromes AND gene expression,” “dry eye syndromes AND microarray analysis,” and “dry eye syndromes AND conjunctiva AND gene expression”.

Most of the rabbit microarray’s top genes (Table 1) have been reported as having some association with DED in humans and/or animal models of the disease [35,36]. These genes include: ORM1, S100A8, S100A9, S10011, IDO1 (each one upregulated) and PIP, LCN2, LTF and lipophilins (each one downregulated) [32,35,37–40]. Moreover, among genes reported as inflammatory markers CXCL8 (IL8), IL1A, IL1B and CCL2 were significantly altered in our DED rabbit model [40,41]. We found that 6 of 10 human DED specific proteins proposed by Zhou et al. [35,41] were significantly changed in our rabbit microarrays (S1 Table).

Two groups of genes merit specific mention, pro-inflammatory genes regulated by interferon and olfactory transcripts. Our findings are summarized below.

#### Interferon-regulated genes correlated with human conjunctival inflammation

We found changes in the following rabbit interferon-regulated genes: CXCL9, CXCL10 and CXCL11, MX1, IRF1, IFI44, IFI44L, IFIT2, IFIT3, IFIT1B, IFRD1, IFI35, IRF7, IFGGA1 (interferon-inducible GTPase 1), LOC100358539 (interferon-induced guanylate-binding protein 1) and LOC100357801 (interferon-induced very large GTPase 1-like protein) [42,43]. Of interest, IFGGA1, LOC100357801, LOC100358539 homologs not previously correlated with DED, may represent candidate biomarkers of IFN-related DED, given the magnitude of their induction by DED (DED vs normal FC = 3.8, 2.2 and 2.3, respectively).

#### Olfactory transcripts

Several rabbit homologues belonging to the family of G-protein coupled receptor (GPCRs) including olfactory receptors (ORs)/odorant binding proteins (OBPs) such as LOC103347214 were changed in the conjunctiva of rabbits with DED. Among 701 olfactory transcripts detected in rabbit conjunctiva tissues, 24 were significantly downregulated and 13 upregulated (FC>1.4, p value < 0.0001) in DED compared to normal (S1 Table). An autoimmune response to odorant binding protein 1a has been associated with DED in the Aire-deficient mouse [44] and identified as a putative autoantigen in a Sjogren’s-like mouse model [46]. Transcriptomic and proteomic profiling of mouse olfactory tissues revealed high proportions of odorant binding and antimicrobial defense proteins, suggested to be a part of a defense mechanism activated by pathogens that regularly enter the body along the ‘eyes-nose-oral cavity’ axis [45].

Table 4 lists 9 genes whose expression is significantly altered in DED compared to normal that have not been previously correlated with DED. Whether these genes play a role in its pathogenesis or can serve as its biomarkers is not clear.

**Table 4:** Putative rabbit DED transcript markers not previously correlated with the disease.

Gene	Name
LOC103347214	odorant-binding protein-like
LOC100008793	lipophilin AS
SCGB1D	lipophilin BL
SCGB1D1	secretoglobin family 1D member 2-like
ENSOCUG00000026544	cytochrome P450 2A11
CYP2A10	cytochrome P450 2A10
ALAS2	5’-aminolevulinate synthase 2
LOC100355142	transmembrane protease serine 11B-like
LOC103347507	major allergen I polypeptide chain 1-like

All these genes displayed ≥2 fold change in their expression in the conjunctiva of rabbits with DED.

### RT-PCR analysis of immune response genes

Inflammation is the core mechanism of DED [7,8]. Activation of the innate and adaptive immune responses in which CD4+ T cells damage the ocular surface and further destabilize an already unstable tear film followed by propagation of the vicious cycle [8,9], which culminates in DED [10,46]. Our microarray results highlight the increased expression of genes associated with immunity induced by rabbit DED (Fig 4), findings consistent with such a pathogenetic model of DED.

Given the centrality of the innate and adaptive immune responses in the development of DED, we confirmed by RT-PCR the changes in the expression of relevant genes. RT-PCR was performed using the Rabbit Innate and Adaptive Immune Responses RT2 Profiler PCR (Qiagen), which evaluates the transcripts of 84 genes involved in them (S1 Table).

**Fig 5:**
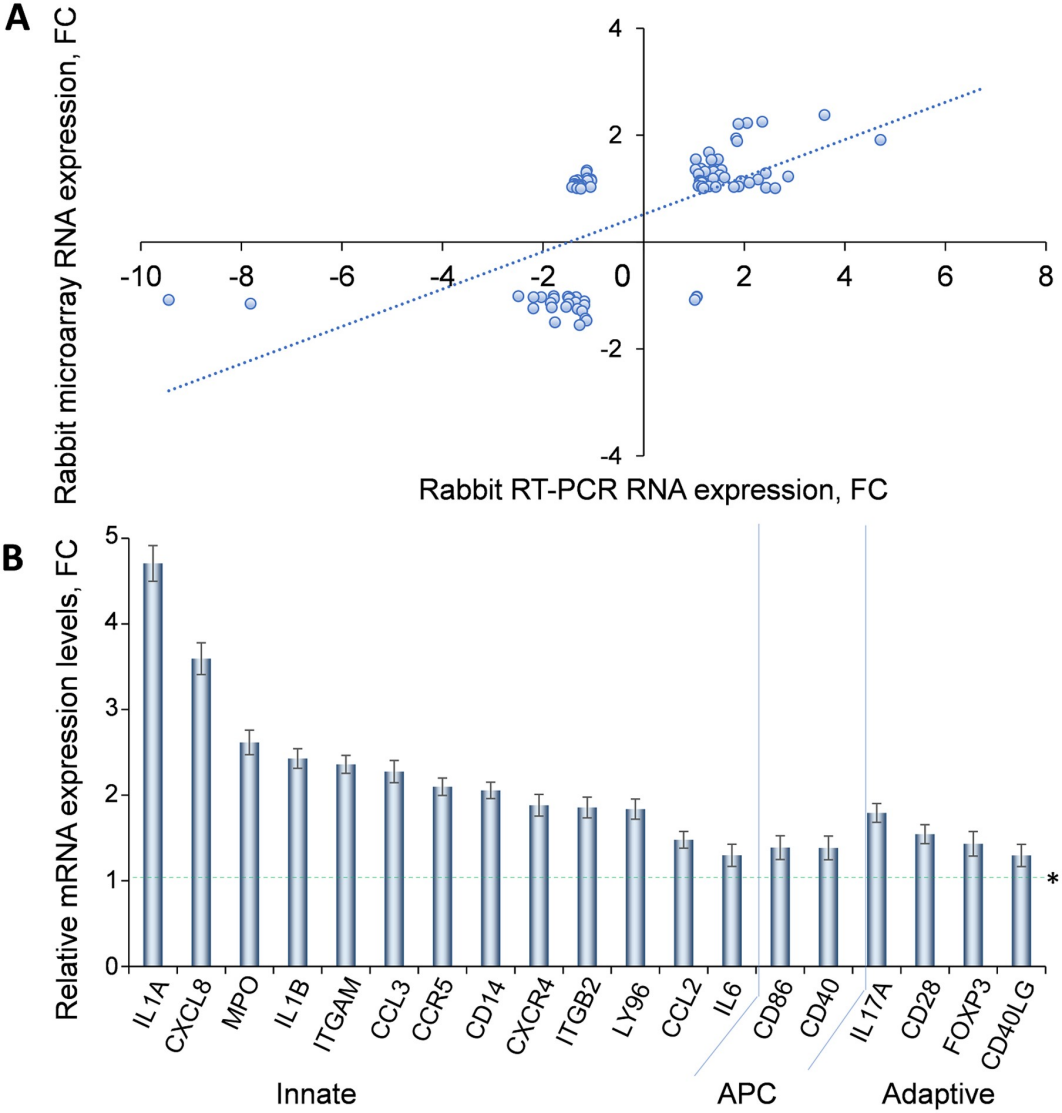
RT-PCR analysis of immune response genes. **A**. Correlation of RNA expression levels of inflammatory mediators in DED rabbits obtained with both: RT-PCR and microarray methods. (PCC R = 0.622, n = 84, p-value < 0.001.) B. The relative expression (RT-PCR) of 18 selected genes representing innate, transition (APC, antigen presenting cells) and adaptive immunity. Each bar represents mean ± SD of fold change (n = 8, *p < 0.001) in DED compared to normal mRNA levels, normalized to the 5 host genes as in Methods.

RT-PCR showed a significant correlation between the 84 genes assayed by RT-PCR and the corresponding genes determined by microarrays (Pearson Correlation Coefficient (PCC) R = 0.622, N = 86, p<0.001, Fig 5A). Fig 5B shows the results on 19 genes selected from those involved in innate and adaptive immunity as well as in antigen presentation. Specifically, IL1A, CXCL8 (IL8), MPO, IL1B, ITGAM, CCL3, CCR5, CD14, CXCR4, ITGB2, LY96, CCL2, IL6 are involved in innate immunity; IL17A, CD28, FOXP3, CD40LG are involved in adaptive immunity; and CD86, CD40 are part of the transition phase characterized by the upregulation of gene transcripts involved in antigen presentation (APC).

**Fig 6:**
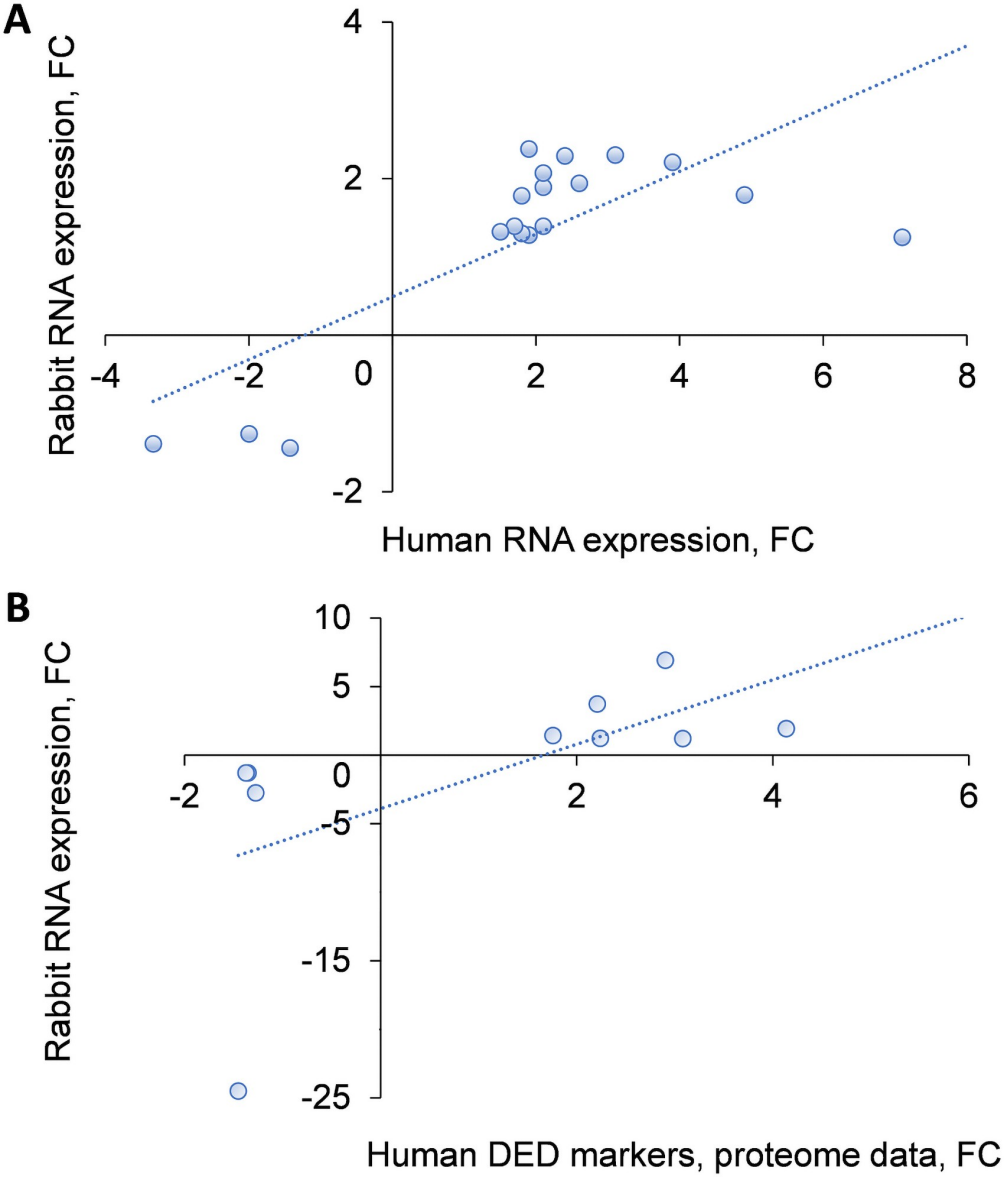
Correlation of gene expression between Sjogren syndrome and rabbits with DED. **A**. Gene expression levels (fold change, FC) in rabbits with DED were correlated with that in DED patients with Sjogren’s syndrome (FC≥1.3 up- or down-regulated, p-value <0.001, n = 18). PCC R = 0.756, p<0.001. B. Similar correlation between rabbit transcripts and human DED protein markers included in this analysis (FC≥1.3, p-value<0.001, n = 9) was found (R = 0.662, p<0.05), as in Methods.

The study of patients with Sjogren’s syndrome evaluated 249 genes [24]. Of them, only 27 were upregulated and 13 downregulated compared to the normal controls; the FC threshold for the upregulated genes was >1.5, and <0.3 for downregulated genes [24]. We analyzed these 40 genes in our microarray data; two of them, *CFB* and *MAFF*, were not present in the rabbit transcriptome. Using as a threshold for gene expression FC >1.25, we found that 23 of the 38 genes were significantly either up- or down-regulated (p<0.001–0.01). Correlation analysis using the Pearson correlation coefficient revealed a statistically significant correlation (R = 0.756; p<0.001) between the differentially expressed genes in the rabbits and in patients with Sjogren’s syndrome (Fig 6A and S1 Table).

Additionally, comparison of transcriptome changes in our rabbits with DED with human proteomics data obtained with iTRAQ technology coupled with 2D-nanoLC-nano-ESI-MS/MS revealed a statistically significant correlation of the 10 DED markers proposed by Zhou et al. [32] (R = 0.662, p<0.05, Fig 6B and S1 Table).

**Fig 7:**
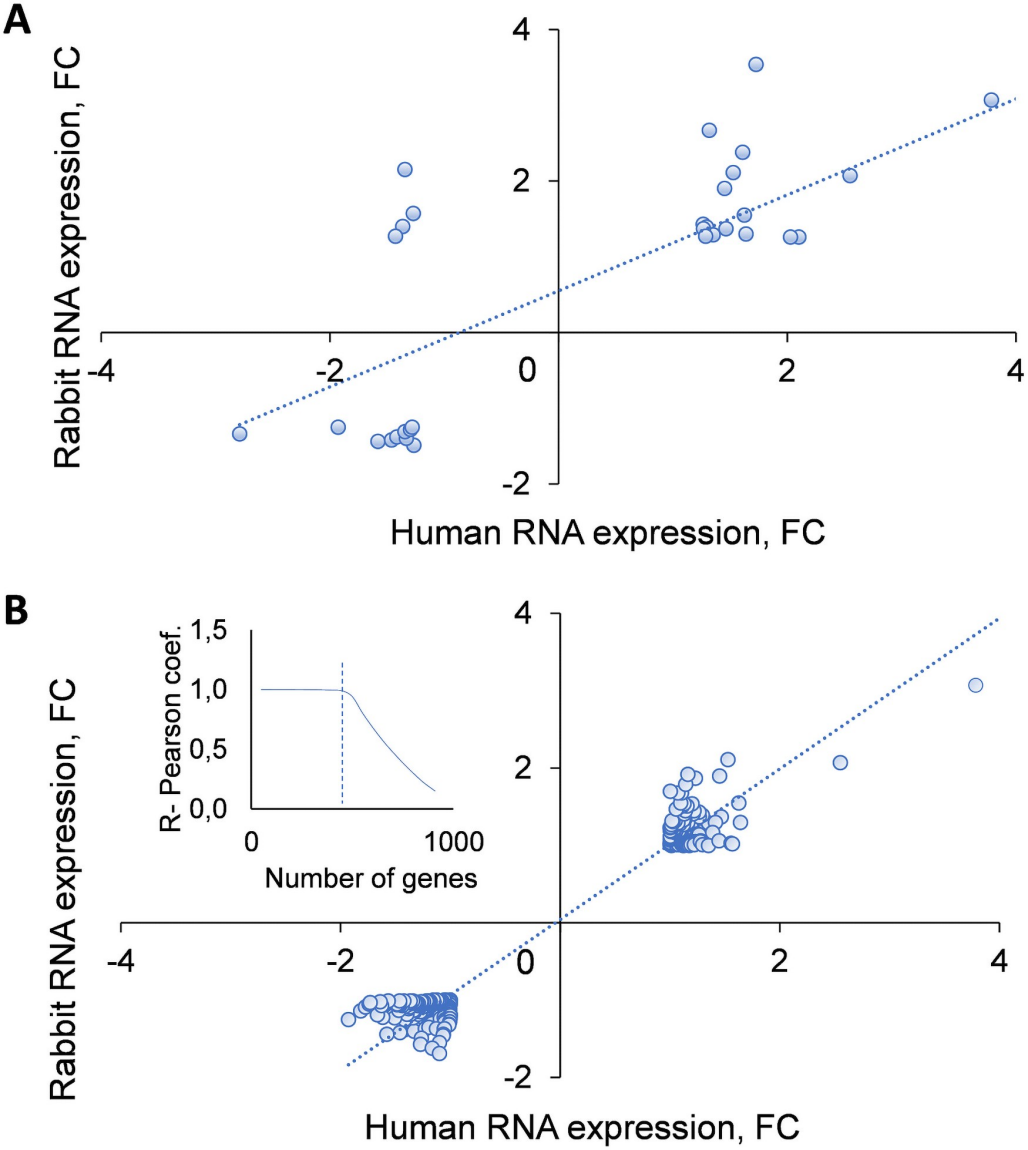
Correlation of gene expression between DED patients without Sjogren syndrome and rabbits with DED. **A.** Gene expression in rabbits with DED was compared to that in patients with non-Sjogren’s syndrome DED. There was a significant correlation between the 32 most changed rabbit and the corresponding human genes included in this analysis (FC≥1.3 up- or down-regulated, PCC R = 0.545; p-value <0.001; *n* = 32). **B**: The expression of 475 of 910 human genes included in this analysis (that were annotated in rabbit transcriptome arrays), was similar in both species (PCC R = 0.917, p<0.0001), as described in Methods. The most correlated genes were cut-off by using the inflection point, determined by the function (small graph on the left) of similarly expressed genes (in both species) and calculated PCC Rs (S1 Table).

The study of gene expression in non-Sjogren’s patients evaluated 1,164 transcripts (NCBI GEO Ac. No: GSE28941) [23]. When we compared them to our 23,364 transcripts, we found 910 fully annotated transcripts common to non-Sjogren’s patients and our rabbits with DED (FC = 1.0; Pearson coefficient r = 0.128; p<0.001). When we applied a threshold of FC ≥1.2 or 1.25 or 1.3 (absolute value) to these 910 genes, we found that 63, 32 and 20 genes, respectively, were significantly correlated between rabbits and human glycosylated proteins (Pearson coefficients r = 0.360, 0.545 and 0.728; p<0.001–0.003, respectively; Fig 7A). Furthermore, the expression of 475 genes out of 910 was similar in the two species. This was determined by calculating the squares of the difference between paired rabbit and human non-Sjogren’s FC gene expression levels (R = 0.917, p<0.0001; Fig 7B; and S1 Dataset Tab D, which also provide the relevant equation).

**Fig 8:**
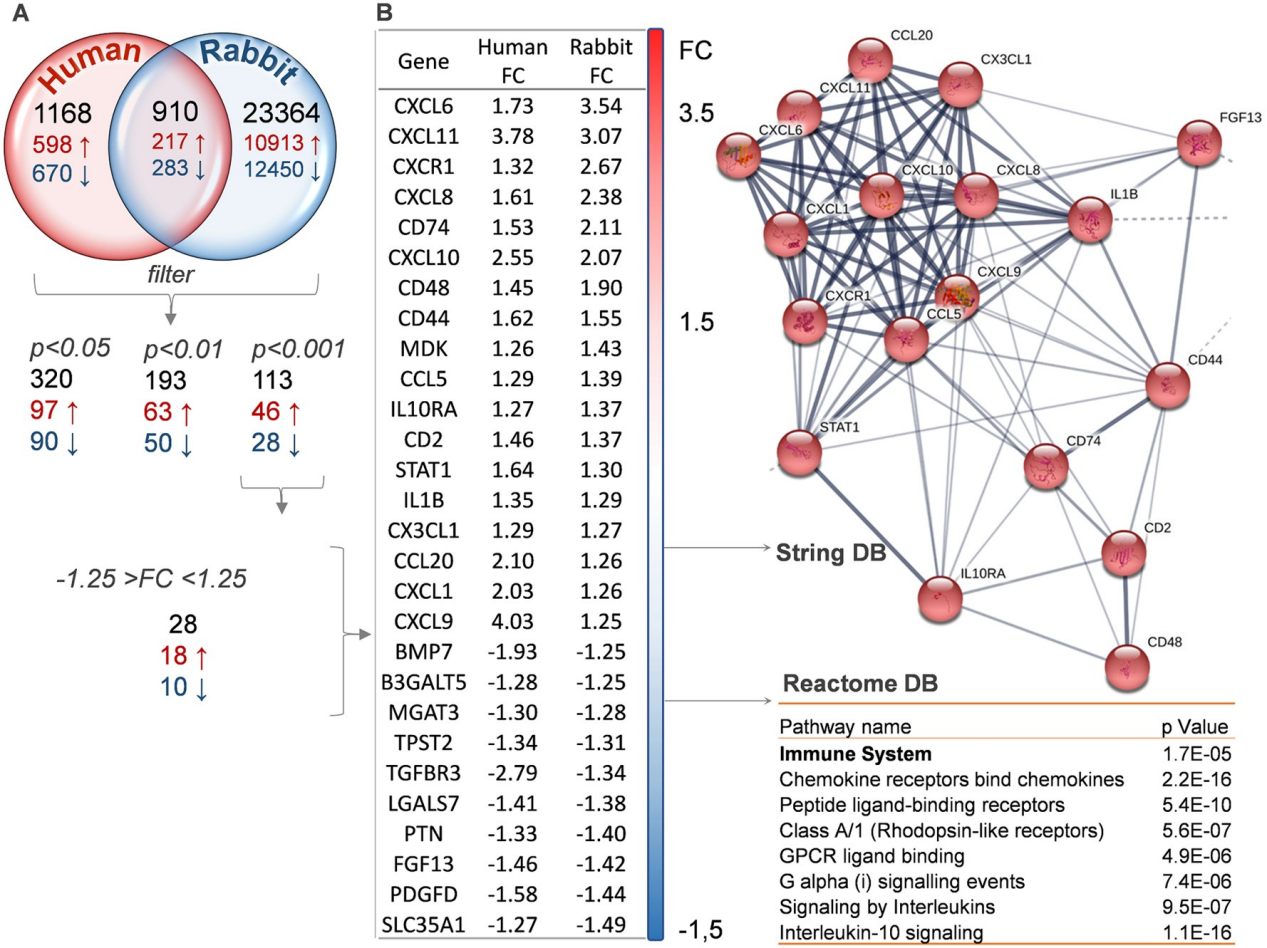
Gene expression in patients with DED not related to Sjogren syndrome. **A**. Venn Diagram depicts the overlap between rabbit and human non-Sjogren’s DED microarray data. Of the 910 overlapping and annotated genes, 217 were similarly upregulated and 283 downregulated in both species, the remaining 410 were changed in opposite directions. The distribution of these genes based on the threshold of statistical significance (TAC, as in Methods) is also shown. **B**. 28 genes were subtracted from the p<0.001 group, based on their FC values and listed in descending order according to the FC in rabbit gene expression. The String database (DB) revealed a cluster among them related to the immune response (upper part), whereas the Reactome DB system identified several pathways, with the top one being the immune system, common to human and rabbit transcriptomes.

As depicted in the Venn diagram (Fig 8), of these 910 genes, 217 are upregulated and 283 downregulated. Using three different p-value thresholds, we defined three subgroups of transcripts, whose total number decreases with increasing statistical significance: 320 for p<0.05, 193 for p<0.01 and 113 for p<0.001 (Fig 8A). When we applied a cut-off of FC >1.25 to the genes in the last subgroup, we identified 28 genes (one quarter of the total) whose expression changed in the same direction (up or down) as in the human transcriptome; the expression of the remaining 4 changed in opposite directions (Fig 8A). Using the String and Reactome databases we found that most of these 28 genes create an immune cluster and also belong to the pathways previously described (Fig 8B, Table 2 and S1 and S2 Datasets).

## Discussion

DED, a common ocular disease, still remains elusive in its detailed pathophysiology, a fact likely contributing to the lack of a successful therapeutic agent. Our comprehensive gene expression analysis of conjunctival tissue in a rabbit model of DED and its comparison to corresponding human data offer a pathophysiological insight into DED that may be relevant to the human disease.

The rabbit is a suitable model for such studies not only because the size of its globe is close to the human but also because of similarities in the histology and biochemistry of the ocular surface. The rabbit DED model we used is robust and has proven useful in the evaluation of candidate drugs. The lectin Concanavalin A, by way of its repeated injections, established chronic DED, akin to the clinically encountered disease. We show that four parameters of DED, which monitor different aspect of the ocular surface, tear composition and stability, were all abnormal and consistent with chronic DED. Principal component analysis of the results, providing a composite of their individual contributions, also confirmed DED [15].

Conjunctiva, a significant portion of the ocular surface, is affected by DED (regardless of its etiology) similar to the cornea. Thus, the molecular changes we observed represent the result of sustained chronic DED at the major target tissue. Moreover, full thickness biopsies should provide a more informative picture of DED, since the inflammatory changes in the ocular surface tissues extend beyond the reach of impression cytology.

Our first observation was that slightly over one-fifth of all rabbit genes are differentially expressed in DED compared to normal controls; of them, roughly half are upregulated and the other half downregulated. This finding suggests long-term and perhaps more complex perturbations in the conjunctiva associated with DED.

Further analysis of these changes with various approaches and focusing on transcripts whose expression was altered in a quantitatively significant manner, unraveled several patterns in these broad changes. Specifically, differentially expressed genes could be segregated into functional modules and clusters; altered pathways; functionally linked genes; and into groups of individual genes of known or suspected pathophysiological relevant to DED. A common feature of all these subgroups is the breadth and magnitude of the changes that encompass essentially all aspects of cell biology and ocular immunology.

The 12 functional modules (7 from the upregulated genes and 5 from the downregulated genes) include such fundamental cell functions as transcriptional and translational control; metabolism of various lipids; prominent intracellular signaling pathways such as Wnt and Notch; and metabolism ranging from the ordinary to drug metabolic pathways. In addition, there are changes that appear to be of greater interest to DED pathophysiology as it is currently understood. They include solute transport genes and aquaporins that may mediate the response to ocular surface desiccation or hyperosmolarity; cytokine signaling likely involved in inflammation; and apoptosis reflecting advanced epithelial damage. Prominent here, as in virtually all types of analysis that we performed, are changes in the expression of genes related to the three components of the immune response, innate and adaptive immunity, and antigen presentation. Cluster analysis also highlighted changes in these immune responses, with changes in cell cycle forming a separate cluster.

The 25 most altered pathways paint a similar picture of potential large-scale changes. Of our five subgroupings of these pathways, 3 are concerned with immune phenomena, namely innate and adaptive immune response and immune cell trafficking and signaling, totaling 10 significantly changed specific pathways. The other two subgroups, encompassing 15 specific pathways, concern gene transcription, translation, and posttranslational modifications. It is worth recalling that this is only a partial listing, as it includes the pathways with the most prominent changes.

Viewed another way, 78 individual genes with quantitatively (and statistically) significant changes represent a broad array of cellular functions along the lines mentioned above, including the immune response. Notable are the S100 genes, known to be involved in DED [34], as well as NOX and HEY genes involved in oxidative stress and Notch and Wnt signaling respectively, all implicated in DED [44].

Two interesting groups of genes whose transcription changed in DED were the multiple genes regulated by type I, II or III interferons and the olfactory genes. The 16 interferon-regulated genes may significantly contribute to ocular surface inflammation [26,37,43]. For example, the inflammatory cycle of DED includes myeloid effector cells such as neutrophils and macrophages which produce cytokines, namely IL-1 and IFNγ that mediate typical epithelial metaplasia and goblet cell loss. Cytokines derived from cellular sources within the conjunctiva induce HLA-DR expression on antigen presenting cells, a function well established to correlate with DED [7,35]. Upregulation of costimulatory molecules such as CD40, CD80, and CD86 involved in APC–T-cell interactions hints at the presence of a yet unidentified autoantigen and the break of tolerance in DED [45]. The perpetuating inflammatory state in DED is dependent on the influx and efflux of T cells from ocular surface to the regional lymph nodes mediated by chemokines and chemokine receptors.

The role of adaptive immunity in DED pathogenesis is well established by disease causing adoptive transfer of CD4+ T cells in nude mice [46] along with presence within the desiccated ocular surface of CD4+ IFNγ and Th17 cells [47]. The latter confer pathogenicity as evidenced by amelioration of DED after in vivo neutralization of IL17 a cytokine associated with epithelial disruption [48,49]. Constant desiccating stress primes epithelial and ocular surface immune cells to generate an innate immune response which in turn and in tandem instructs sentinel adaptive immune events leading to typical DED pathologic changes.

The olfactory genes may be part of the ‘eyes-nose-oral cavity’ axis speculated to participate in DED. Indeed, an autoimmune response to odorant binding protein 1a has been associated with DED in the Aire-deficient mouse and identified as a putative autoantigen in a Sjogren’s-like mouse model [50]. Transcriptomic and proteomic profiling of mouse olfactory tissues revealed high proportions of odorant binding and antimicrobial defense proteins, suggested to be a part of a defense mechanism activated by pathogens that regularly enter the body through the facial ports of entry (eyes, nose and mouth) [50].

Changes in the transcription of immune response genes are ubiquitous, as confirmed in our microarray data by RT-PCR. Analysis of the transcripts of a well-selected group of 84 relevant genes revealed a strong correlation between the two methods, validating the microarray results. These genes cover multiple participants in the immune response.

There are two important questions regarding our findings: Their degree of similarity to human DED, and their relevance to the development of the disease. The answers to both are, out of necessity, partial.

Human DED transcriptomic studies are limited by the low number of transcripts analyzed [26,42,48]. Nevertheless, the two most extensive studies allowed a comparison between human and rabbit transcriptomes revealing a significant correlation between them. It was interesting that one study concerned Sjogren’s patients, considered the prototypical aqueous-deficient form of DED [24] and the other non-Sjogren’s patients, presumably representing either evaporative or mixed DED [23]. [One potential corollary to this observation is that at the transcriptome level the pathophysiological subtypes may be quite similar if not identical. This is in line with evidence suggesting that conjunctival histopathologic features in DED patients are similar between Sjogren’s and non-Sjogren’s patients [45]. It is worth recalling that absent the history and associated clinical findings, the etiopathogenesis of DED cannot be identified by examination of the ocular surface or tears.

**Fig 9:**
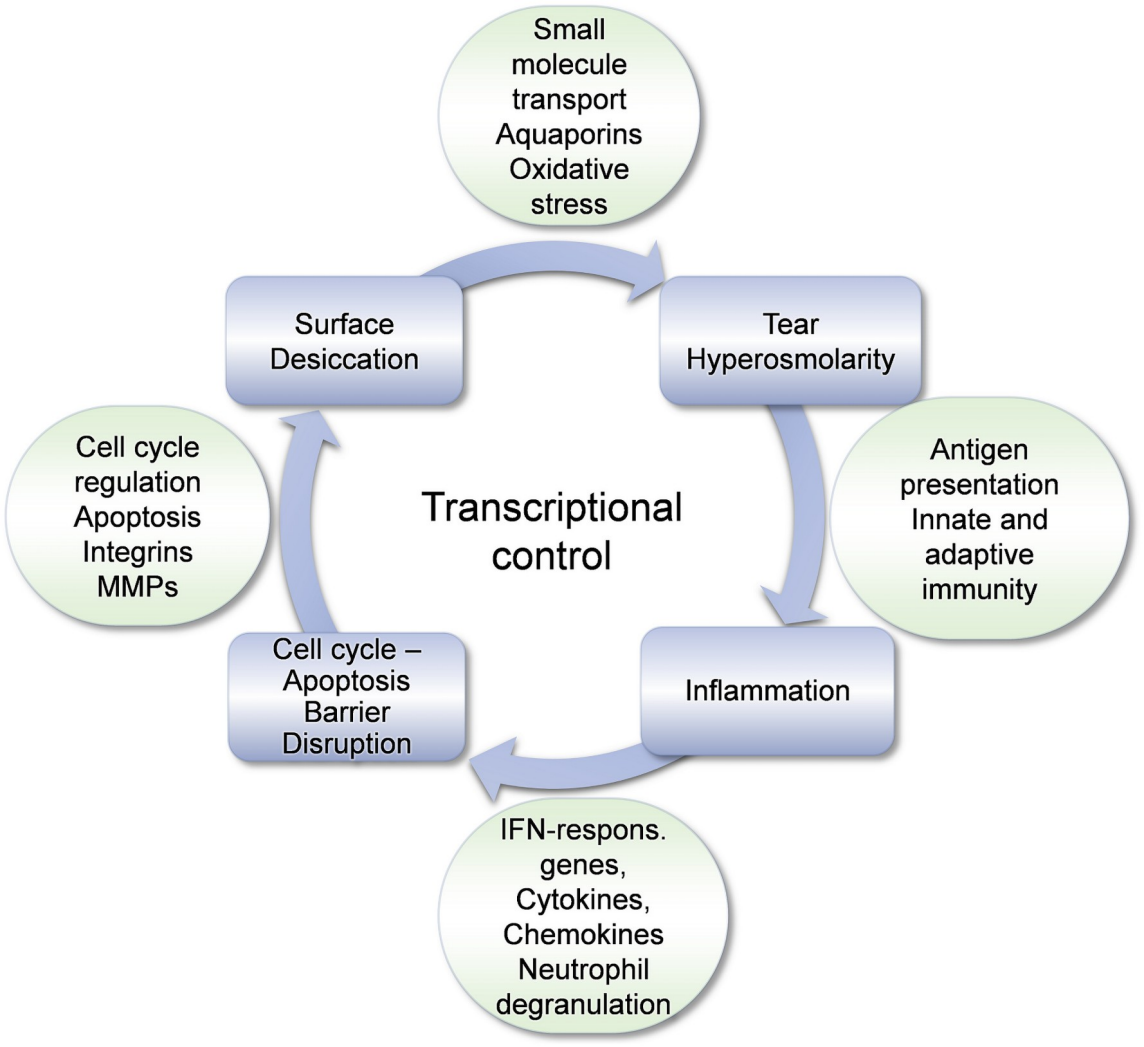
Transcriptome changes during the vicious cycle of DED. The four components of the vicious cycle of the pathophysiology of DED are shown. Representative changes in gene expression observed in rabbits with DED are linked to each stage of the cycle. Changes in genes involved in transcriptional control (depicted at the center of the cycle) likely affect all stages of DED evolution.

A synthesis of these data was attempted in order to relate them to the pathophysiology of DED. even though such an effort has its limitations. For example, our data, extensive as they may be, reflect a single time point in the evolution of the disease, and thus it is difficult to assign the temporal sequence of changes that could suggest etiological associations. Such limitations notwithstanding, we selected transcriptomic changes based on their magnitude, consistency (identified by more than one types of analysis) or pathophysiological plausibility based on extant knowledge. Such changes could be viewed against the “vicious cycle” of DED, which conceptualizes its pathophysiology (Fig 9). Thus, several pathways e.g., cytokine signaling; families of effector molecules that contribute to the development of DED, e.g., aquaporins and MMPs; or broader cellular processes, e.g., cell cycle control can be linked to individual components of the DED cycle.

The expression of numerous genes or families of genes participating in transcriptional control was also altered in our study (Table 2). It is, however, impossible to identify the DED stage that each of the 19 entries under the subheading “Gene transcription” in Table 2 participates. Given the involvement of transcriptional regulation in numerous cellular processes, we suspect that transcriptional control may participate in all stages of the DED cycle; this consideration dictated its position at the center of the cycle in Fig 9.

It would be very important to identify which of these changes are *essential* for the development of DED. We speculate that only some, perhaps few, of these changes initiate DED while a larger number of them, likely not all of them, contribute to its clinical manifestations.

Interestingly, our finding of so pervasive molecular changes associated with DED, at least in its chronic stage, which corresponds to its routine clinical presentation, may explain the difficulties in developing successful treatments for this disease [1]. For example, efforts narrowly targeting one or a small range of signaling pathways may not be sufficient to overcome some of the many parallel or redundant pathways perturbed in DED. If this speculation is borne out by further work, therapeutic strategies may well favor the development of multi-targeted agents.

A practical consideration is that the rabbit model that we used recapitulates not only the signs and symptoms of the human disease but also the molecular changes underlying DED. Our findings suggest that this model is suitable for the study of the pathogenesis of DED, regardless of its subtype, as there appears to be a common final molecular platform once the disease is established. As already mentioned, this model (and its earlier versions) has been useful in the assessment of DED therapeutics [11].

## Conclusions

Our findings provide strong evidence of large-scale changes in the expression of over a fifth of the rabbit genome in the conjunctiva associated with chronic DED. These changes encompass vital cellular functions and are not random in nature, as evidenced by specific alterations in over 25 key signaling pathways and genes controlling the immune response of the ocular surface. There is significant correlation between rabbit and human DED, both aqueous-deficient and evaporative. Several of the observed changes are consistent with current understanding of DED pathophysiology but the specific contribution of each of the multiple changes remains uncertain. Our data offer new and significant insights to our understanding of the pathophysiology of DED and to the future development of successful treatments.

## Supporting information

S1 Fig Protein association networks in DED. Functional enrichment analysis of protein association networks (determined by String) in rabbits with DED induced as in Methods.
(PDF)

S1 Table Reactome analysis of gene expression in rabbits with DED. Analysis of pathway knowledge (Reactome) of differentially expressed genes in rabbits with DED induced as in Methods.
(PDF)

S1 Dataset Gene expression in human and rabbit DED. Gene expression data from rabbits with DED obtained by microarray analysis and RT- PCR) and from humans as reported in the literature (referenced in the text).
(XLSX)

S2 Dataset Pathway analysis in DED. The DED rabbit transcriptome was analyzed by String to identify clusters and then subjected to Reactome analysis.
(XLSX)
