# Supplementary material for: The transcriptome of rabbit conjunctiva in dry eye disease: Large-scale changes and similarity to the human dry eye
Source: PLoS One. 2021 Jul 29;16(7):e0254036. doi: 10.1371/journal.pone.0254036 (PMC8321226; doi:10.1371/journal.pone.0254036)
Supplement: S1 Fig — Functional enrichment analysis of protein association networks (determined by String) in rabbits with DED induced as in Methods. (PDF) [file pone.0254036.s001.pdf]

Supplemental Figure 1. Cluster analysis of the differentialy expressed genes

The cluster analysis of the differentialy expressed genes with FC>1.5-fold $p<0.05$ (upregulated, **A**) or FC<-1.25-fold $p<0.05$ (downregulated, **B**) was performer with the String database (https://string-db.org) Algorithm used: MCL. Inflation parameter = 1.9. Reference orgamism: *Oryctolagus cuniculus* (rabbit).

A

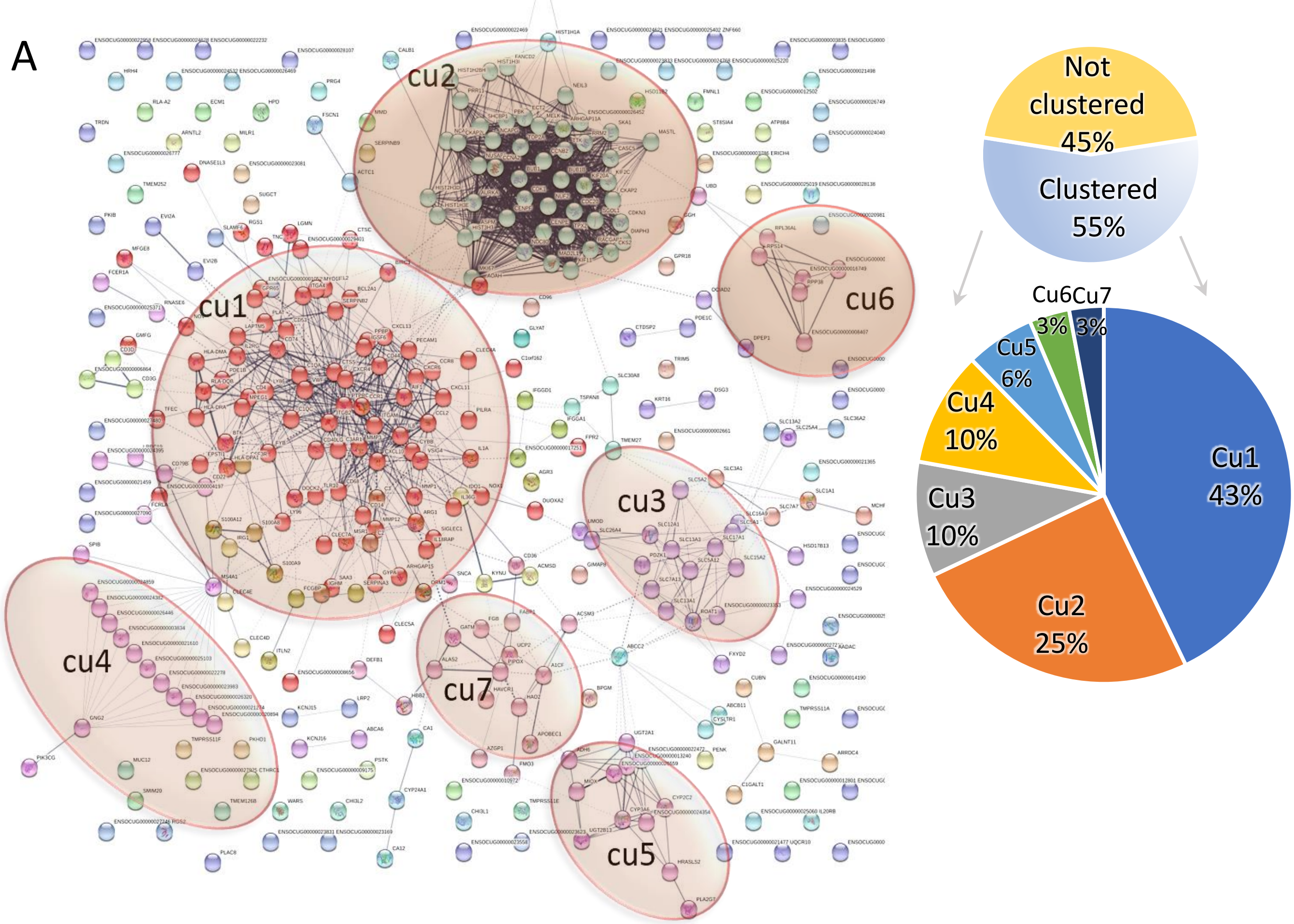

Cluster	Gene no.	Gene %	Upregulated genes
Cu1	87	23.3	Innate and adaptive immune response, chemokines, GPCRs, TLRs, MHC class II antigen presentation MHC II
Cu2	51	13.8	Cell Cycle, cell cycle checkpoints, mitotic Metaphase and Anaphase, kinetochores
Cu3	20	5.4	SLC-mediated transmembrane transport: SLC5A2, SLC5A1, SLC26A4, ABC transporters
Cu4	20	5.4	G proteins, signalling through PI3Kg, PLCb, prostacyclins, potassium channels, aquaporins, GABA B receptors
Cu5	12	3.3	Metabolism of drugs, oxidation, glucuronidation, Phase I and II - functionalization of compounds, PPARA, regulation of lipid and phospholipid metabolism
Cu6	7	1.9	Translational control (initiation, elongation, termination), ribosome processing, selenocysteine synthesis, SRP targeting, Nonsense Mediated Decay (NMD), rRNA processing, mRNA editing
Cu7	6	1.6	Beta defensins, antimicrobial peptides, metabolism of amino acids and derivatives
Tested genes (total)	512	100.0	
Found in String database	369	72.1	
Clustered	203	55.0	

B

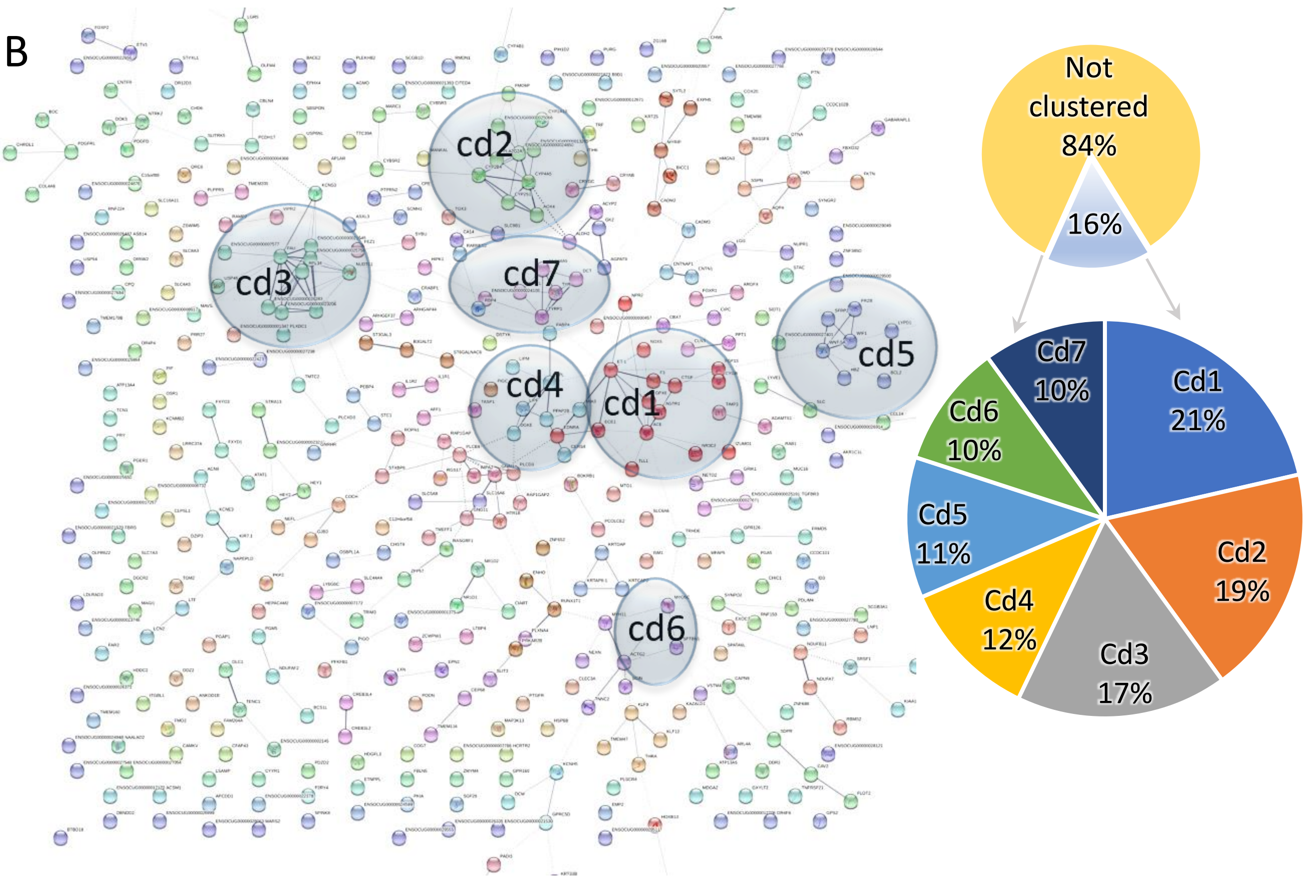

Cluster	Gene no.	Gene %	Downregulated genes
Cd1	17	21	Transcriptional contol: nuclear receptors, cellular responses to stress and external stimuli, detoxification of ROS, A/1 rhodopsin-R
Cd2	16	19	Translational control (elongation, termination), 40S ribosome turnover, Selenocysteine synthesis, SRP targeting, Nonsense Mediated Decay (NMD)
Cd3	14	17	Metabolism, phase I - functionalization of compounds, oxidations, immune response, TNF signaling, death receptor signalling, sulfide oxidation to sulfate
Cd4	10	12	Lipid and sphingolipid metabolism, adipocyte differentiation, triglyceride catabolism, GPCR
Cd5	9	11	WNT, WNT5, FZD4, DVL, TCF signaling, estrogen signaling, intristic apoptosis, inactivate anti-apoptotic BCL-2 members, IL-4 and IL-13, DVL, YAP1-mediated transcription, inositol metabolism
Cd6	8	10	Cytoskeleton and signal transduction, ankyrins, muscle contraction, axon guidance, Ca-dependent events, FOXO-mediated transcription, oxidative stress, transport to the Golgi
Cd7	8	10	Aminoacid and small molecules transport and metabolism, melanin biosynthesis, defective SLC24A5, transport of small molecules, amino acids metabolism
Tested genes (total)	698	100.0	
Found in String database	518	74.2	
Clustered	83	16.0	
