# Supplementary material for: The transcriptome of rabbit conjunctiva in dry eye disease: Large-scale changes and similarity to the human dry eye
Source: PLoS One. 2021 Jul 29;16(7):e0254036. doi: 10.1371/journal.pone.0254036 (PMC8321226; doi:10.1371/journal.pone.0254036)
Supplement: S1 Table — Analysis of pathway knowledge (Reactome) of differentially expressed genes in rabbits with DED induced as in Methods. (PDF) [file pone.0254036.s002.pdf]

Supplemetary tables S1

Table S1.A. Differentially expressed genes sorted in the most affected pathways (Reactome).

	Gene	***Fold change***	***p-value***
**Innate immunity**			
Neutrophil degranulation	S100A12	8.49	2.9e-14
	CHI3L1	3.89	3.2e-10
	S100A8	3.72	1.0e-10
	CXCR1	2.67	4.7 e-9
	CYBB	2.31	4.9 e-8
	CXCR2	2.28	5.0 e-8
	ITGAM	2.25	5.5 e-8
	CD14	2.23	5.8 e-10
	CLEC4D	2.22	7.7 e-7
	PPBP	2.16	2.0 e-4
	PTPRC	2.11	4.7 e-10
	FPR2	1.99	0.002
	PLAC8	1.99	2.4 e-8
	MS4A1	1.94	2.0 e-4
	S100A9	1.93	4.2 e-8
	ITGB2	1.89	2.2 e-8
	CD68	1.88	1.3 e-9
	CD53	1.86	2.0 e-9
	CD36	1.82	3.1 e-5
	PECAM1	1.80	1.2 e-6
	SERPINB2	1.73	1.1 e-7
	NCKAP1L	1.71	6.6 e-7
	CLEC5A	1.70	0.001
	C3AR1	1.70	1.9 e-5
	CTSS	1.70	1.0 e-8
	CTSC	1.66	8.4 e-8
	GMFG	1.65	4.0 e-6
	ARG1	1.63	3.0 e-6
	DOCK2	1.61	4.0 e-6
	GGH	1.60	2.0 e-4
	TCN1	-2.26	1.0 e-6
	OLFM4	-1.9	9.3 e-7
	PTPRN2	-1.71	2.5 e-5
Trafficking and processing of endosomal TLRs	LY96	1.94	4.3 e-9
	TLR10	1.63	0.005
	LY86	1.61	1.0 e-4
**Adaptive immunity**			
MHC class II antigen presentation	HLA-DQA1	3.17	9.2e-12
	HLA-DRB1	2.38	6.5e-4
	HLA-DRA	2.29	1.9e-10
	HLA-DMA	1.88	2.3e-7
	CD74	2.11	1.2 e-9
	KIF11	1.84	7.1 e-7
	HLA-DOA	1.78	2.9 e-7
	KIF20A	1.71	1.6 e-7
	CTSS	1.70	1.0 e-8
	CTSC	1.66	8.4 e-8
	RACGAP1	1.61	1.7 e-5
	CD4	1.55	0.0005
Translocation of ZAP-70 to Immunological synapse	CD3D	1.91	2.0 e-6
	CD3G	1.74	2.0 e-6
	CD4	1.55	0.0005

TCR signaling	CD3D<br>FYB<br>CD3G<br>CD4	1.91<br>1.86<br>1.74<br>1.55	2.0 e-6<br>5.8 e-5<br>2.0 e-6<br>0.0005
Downstream TCR signaling	CD3D<br>CD3G<br>CD4	1.91<br>1.74<br>1.55	2.0 e-6<br>2.0 e-6<br>0.0005
Phosphorylation of CD3 and TCR zeta chains	CD3D<br>CD3G<br>CD4	1.91<br>1.74<br>1.55	2.0 e-6<br>2.0 e-6<br>0.0005
Generation of second messenger molecules	CD3D<br>FYB<br>CD3G<br>CD4	1.91<br>1.86<br>1.74<br>1.55	2.0 e-6<br>5.8 e-5<br>2.0 e-6<br>0.0005
PD-1 signaling	CD3D<br>CD3G<br>CD4	1.91<br>1.74<br>1.55	2.0 e-6<br>2.0 e-6<br>0.0005
Interaction between lymphocytes and antigen presenting cells	CD96	1.57	0.0013
**Cytokine signaling**			
Interleukin 10 signaling	CXCL10<br>FPR2<br>IL1A<br>CCR1<br>ALDH2<br>MARC1<br>PADI3<br>LY6G6C<br>LSAMP<br>PLCB4<br>DOK5<br>RAMP2<br>CPE<br>NEFL<br>RSPO3<br>NAPEPLD	2.07<br>1.99<br>1.91<br>1.79<br>-1.70<br>-1.67<br>-1.67<br>-1.63<br>-1.63<br>-1.63<br>-1.63<br>-1.63<br>-1.60<br>-1.60<br>-1.59<br>-1.58	2.4 e-7<br>0.002<br>1.0 e-4<br>1.6 e-5<br>2.2 e-6<br>4.5 e-8<br>1.6 e-6<br>6.8 e-8<br>3.1 e-7<br>1.0 e-6<br>1.2 e-5<br>0.001<br>3.1 e-5<br>2.0 e-4<br>0.004<br>1.0 e-6
Interleukin 12 signaling	CA1	2.77	1.0 e-7
Interleukin 1 signaling	IL18RAP<br>IL36G<br>CASP1	1.51<br>1.51<br>1.21	0.013<br>0.010<br>2.0 e-4
Interleukin 4 and 13 signaling	LCN2	-2.03	1.0 e-6
**Signal transduction**			
Chemokine receptors bind chemokines	CXCL6<br>CXCL11<br>CXCL8<br>CCL7<br>CXCR4<br>CXCL10<br>CXCL13<br>CCR1<br>CCRL2<br>CXCR6	3.54<br>3.07<br>2.38<br>2.37<br>2.21<br>2.07<br>1.92<br>1.79<br>1.72<br>1.54	8.7 e-11<br>7.0 e-8<br>1.5 e-8<br>4.5 e-8<br>1.1 e-6<br>2.4 e-7<br>1.0 e-6<br>1.6 e-5<br>1.2 e-7<br>8.5 e-5
Signaling by NOTCH4	HEY1<br>HEY2	-1.63<br>-1.62	4.0 e-6<br>5.5 e-5
Signaling by Rho-GTPase	NOX1<br>RACGAP1	2.30<br>1.61	6.3 e-9<br>1.7 e-5
Signaling by GPCR	GPR18	2.25	3.0 e-6

	HTR1B	-2.36	4.0 e-7
	OPN5	-1.91	1.9 e-7
	CCL21	-1.87	7.7 e-9
	OR5W2	-1.72	0.016
	GPRC5A	-1.69	3.1 e-7
	PLCB4	-1.63	1.0 e-6
	RAMP2	-1.63	0001
Signaling by BCR	CD79B	1.6	4.0 e-6
Signaling by Wnt	WIF1	-2.0	3.6 e-8
**Gene expression**			
Nuclear Receptor transcription pathway	NR1D1	-2.01	1.9 e-9
Transcriptional regulation by RUNX3	ITGAM	2.25	5.5 e-8
	ITGA4	1.79	2.7 e-5
	CD4	1.55	0.0005
	NKX3-1	-1.89	2.1 e-7
	STAC	-1.87	0.0004
	HEY1	-1.63	4.0 e-6
	HEY2	-1.62	5.5 e-5
	RSPO3	-1.59	0.004
Oxidative stress mediated transcription	ABCA6	2.77	1.43 e-7
	FBXO32	-1.53	0.001
**Extracellular matrix organization**			
Integrin cell surface interactions	MMP12	3.06	3.7 e-8
	MMP3	1.56	1.0 e-6
	MMP1	1.51	4.3 e-5
	MMP13	1.29	0.002
	MMP9	1.17	0.039
Degradation of extracellular matrix	ADAMTS1	-1.58	1.0 e-6
	CAPN9	-1.51	1.0 e-6
**Metabolism of RNA**			
Nonsense Mediated Decay (NMD) independent of the Exon Junction Complex (EJC)	NOV	1.95	7.8 e-9
	RPS14	1.64	2.0 e-6
	BTBD18	-1.59	0.042
**Protein translation**			
Peptide chain elongation	RPS14	1.64	2.0 e-6
	RPL34	-1.84	1.84 e-7
Post translation modification	VNN3	-4.69	2.4 e-12
	LYPD1	-3.69	1.0 e-9
	PGAP1	-1.84	0.0001
	LY6G6C	-1.63	6.8 e-8
	LSAMP	-1.63	3.1 e-7
**Cell cycle**			
Polo-like kinase mediated events	CCNB2	2.11	2.3 e-8
	CENPF	1.67	4.0 e-6
Cell cycle checkpoints	CDK1	2.48	5.8 e-8
	KIF2C	1.59	3.5 e-5
**Apoptosis**	CASP3	1.44	5.9 e-5
	CASP10	1.37	7.0 e-4
	CASP1	1.21	2.0 e-4
**Axon guidance and cell migration**			
Regulation of expression of SLITs and ROBOs	RPL36AL	2.39	1.1 e-5
	RPS14	1.64	2.0 e-6
	RACGAP1	1.61	1.7 e-5
	CENPE	1.58	7.0 e-6
	MAD2L1	1.58	2.4 e-7

**Metabolism**			
O-linked glycosylation	MUC12<br>MUC16<br>MUC5AC	2.87<br>-1.62<br>-1.21	8.0 e-8<br>2.0 e-6<br>0.009
Keratinization	KRT16<br>KRT18<br>SPRR3<br>TCHH<br>PKP2	2.44<br>-2.42<br>-2.03<br>-1.87<br>-1.53	1.0 e-6<br>2.4 e-8<br>7.3 e-8<br>0.003<br>9.0 e-6
Transport of small molecules	SLC13A1<br>SLC5A12<br>SLC17A1<br>SLC3A1<br>SLC16A9<br>SLC36A2<br>SLC15A2<br>SLC7A13<br>SLC5A2<br>SLC13A3<br>SLC30A8<br>TMEM252<br>SCGB1D1<br>SLC7A3<br>SLC4A5<br>SLC5A8<br>SLC24A5<br>ATP13A5	2.30<br>2.0<br>2.0<br>1.94<br>1.92<br>1.89<br>1.68<br>1.66<br>1.65<br>1.64<br>1.63<br>1.59<br>-6.9<br>-2.17<br>-1.78<br>-1.72<br>-1.67<br>-1.59	2.2 e-9<br>4.4 e-9<br>1.0 e-6<br>4.3 e-8<br>2.1 e-7<br>1.0 e-5<br>1.0 e-6<br>1.7 e-5<br>1.6 e-6<br>2.0 e-6<br>2.0 e-6<br>2.0 e-6<br>6.2 e-9<br>9.0 e-6<br>2.0 e-6<br>3.3 e-6<br>6.1 e-5<br>4.4 e-7
Oxidation	NOX1<br>GSTA2<br>PIPOX<br>HAO2<br>GPX3<br>AOX4	2.30<br>2.26<br>1.63<br>1.60<br>1.54<br>-1.75	6.3 e-9<br>1.2 e-8<br>4.0 e-4<br>1.0 e-6<br>0.0003<br>9.0 e-6
Hydroxylation	CYP4B1	-1.60	2.0 e-6
Regulation of IGF transport	PENK	1.94	1.0 e-6
**Miscellaneous**			
Antimicrobial peptide	DEFB1<br>LTF	1.60<br>-2.76	3.0 e-6<br>5.3 e-9
Transmission across electrical and chemical synapses	PANX2<br>FLOT2<br>GRIK1<br>RASGRF1<br>NEFL	-2.09<br>-1.81<br>-1.72<br>-1.65<br>-1.60	3.2 e-9<br>0.01<br>0.006<br>2.0 e-6<br>2.0 e-4
Neurotransmitter clearance	ALDH2	-1.70	2.2 e-6
Water transport	AQP4	-1.65	2.0 e-5
Cellular response to heat stress	CRYAB	-1.51	3.9 e-7

Table S1.B. Differentially expressed genes common to rabbit and patients with Sjogren's syndrome.

Official gene symbol (NCBI Gene)	Human*	Rabbit	p-value
CXCL9	7.10	1.25	0.01185
CCR1	4.90	1.79	0.00002
CXCR4	3.90	2.21	0.00000
NOX1	3.10	2.30	0.00000
LY96	2.60	1.94	0.00000
HLA-DRA	2.40	2.29	0.00000
ITGB2	2.10	1.89	0.00000
CXCL10	2.10	2.07	0.00000
NOS2	2.10	1.39	0.00120
HLA-DRB1	1.90	2.38	0.00007
C1QB	1.90	1.28	0.00006
STAT1	1.80	1.30	0.00001
C2	1.80	1.78	0.00000
CCL5	1.70	1.39	0.00002
IL15	1.50	1.32	0.01600
PTGFR	-3.33	-1.39	0.00090
GNGT1	-2.00	-1.26	0.01122
HMGB2	-1.43	-1.44	0.00006

*Literature: H. Liang, K. Kessal, G. Rabut, P. Daull, J.S. Garrigue, S. Melik Parsadaniantz, M. Docquier, C. Baudouin, F. Brignole-Baudouin, Correlation of clinical symptoms and signs with conjunctival gene expression in primary Sjogren syndrome dry eye patients, Ocul. Surf. 17 (2019) 516–525. https://doi.org/10.1016/j.jtos.2019.03.005.
https://www.ncbi.nlm.nih.gov/pubmed/30905840

Table S1.C. Differentially expressed human DED related genes and potential DED biomarkers.

Rabbit (Oryctolagus cuniculus) gene expression (microarray data), sorted according to FC and p-value (Transcriptome Analysis Console) for cluster analysis of rabbit.

Gene name	Gene full name	Gene Accession no.	Human*	Rabbit	p-value
PIP	prolactin-induced protein PIP	NM_001171116	-1.45	-24.52	5.49E-14
ORM1	orosomucoid 1	NM_001101695	2.90	6.91	1.41E-12
S100A8	S100 calcium binding protein A8	XM_002715343	2.21	3.72	1.05E-10
LTF	lactotransferrin	XM_008260521	-1.27	-2.76	5.31E-09
S100A9	S100 calcium binding protein A9	NM_001256473	4.13	1.93	4.21E-08
LYZ	lysozyme C	XM_002711324	-1.35	-1.30	4.10E-03
S100A4	protein S100-A4	XM_008264410	3.08	1.19	1.44E-02
S100A11	S100 calcium binding protein A11	ENSOCUT00000005200	1.76	1.43	5.70E-03
ENO1	enolase 1, (alpha)	XM_002716143	2.24	1.21	8.27E-01
LCN1	lipocalin-1-like	XM_008253095	-1.37	-1.31	9.96E-01
S100A12	S100 calcium binding protein A12	XM_002715516	no data	8.40	3.28E-11
LCN2	lipocalin 2	XM_002722973	no data	-2.03	1.17E-06

*Literature: L. Zhou, R.W. Beuerman, C.M. Chan, S.Z. Zhao, X.R. Li, H. Yang, L. Tong, S. Liu, M.E. Stern, D. Tan, Identification of tear fluid biomarkers in dry eye syndrome using iTRAQ quantitative proteomics, J Proteome Res. 8 (2009) 4889–4905. https://doi.org/10.1021/pr900686s.

https://pubs.acs.org/doi/pdf/10.1021/pr900686s

https://pubs.acs.org/doi/suppl/10.1021/pr900686s/suppl_file/pr900686s_si_001.pdf

Table S1.D. Differentially expressed genes common to rabbit and patients with Non-Sjogren's syndrome.

Official gene symbol (NCBI Gene)	Human*	Rabbit	p-value
CXCL9	4.03	1.25	0.000119
CXCL11	3.78	3.07	0.000000
CXCL10	2.55	2.07	0.000000
CCL20	2.10	1.26	0.000228
CXCL1	2.03	1.26	0.000106
CXCL6	1.73	3.54	0.000000
STAT1	1.64	1.30	0.000007
CD44	1.62	1.55	0.000001
CXCL8	1.61	2.38	0.000000
CD74	1.53	2.11	0.000000
CD2	1.46	1.37	0.000331
CD48	1.45	1.90	0.000000
IL1B	1.35	1.29	0.001000
CXCR1	1.32	2.67	0.000000
CCL5	1.29	1.39	0.000016
CX3CL1	1.29	1.27	0.000700
IL10RA	1.27	1.37	0.000114
MDK	1.26	1.43	0.000300
SLC35A1	-1.27	-1.49	0.000068
GALNT11	-1.27	1.57	0.000001
B3GALT5	-1.28	-1.25	0.008041
MGAT3	-1.30	-1.28	0.000260
PTN	-1.33	-1.40	0.000011
CLEC7A	-1.34	2.15	0.000000
TPST2	-1.34	-1.31	0.000300
B3GNT5	-1.36	1.40	0.000100
LGALS7	-1.41	-1.38	0.000027
HPSE	-1.43	1.27	0.000215
FGF13	-1.46	-1.42	0.000316
PDGFD	-1.58	-1.44	0.000001
BMP7	-1.93	-1.25	0.000270
TGFBR3	-2.79	-1.34	0.000008

*Literature: F. Mantelli, L. Schaffer, R. Dana, S.R. Head, P. Argueso, Glycogene expression in conjunctiva of patients with dry eye: downregulation of Notch signaling, Invest. Ophthalmol. Vis. Sci. 50 (2009) 2666–72. https://doi.org/10.1167/iovs.08-2734.
https://www.ncbi.nlm.nih.gov/geo/query/acc.cgi?acc=GSE28941
The human microarray data of conjunctival epithelium of patients with severe DED, deposited as GSE28941 DataSets in NCBI's Gene Expression Omnibus (GEO), were analyzed with GEO2R software provided by NCBI GEO and correlated with our rabbit microarray data.

Table S1.E. Differentially expressed rabbit transcripts encoding olfactory receptors.

Gene	Gene name	FC,<br>p-value<0.001
LOC103347214	odorant-binding protein-like receptor	-18.52
LOC100339545	olfactory receptor 2G6-like	-2.03
LOC100344041	olfactory receptor 5M3-like	-1.99
LOC100340981	olfactory receptor 502-like	1.87
LOC100348626	olfactory receptor 51A4-like	1.83
LOC100343942	olfactory receptor 1F1-like	-1.73
LOC100356089	olfactory receptor 1002-like	-1.72
LOC100337866	olfactory receptor 2AJ1-like	1.68
LOC100355911	olfactory receptor 6F1-like	1.64
LOC100350606	olfactory receptor 6C4-like	-1.64
LOC100340632	olfactory receptor 6-like	-1.64
LOC100355681	olfactory receptor 10V1	1.6
LOC100347565	olfactory receptor 6B1	1.6
LOC100344026	olfactory receptor 7A17-like	1.57
LOC100358408	olfactory receptor 4C12-like	-1.57
LOC100349050	olfactory receptor 2T29-like	-1.57
LOC100354154	olfactory receptor 8G1-like	-1.56
LOC103352009	olfactory receptor 5B2-like	1.56
LOC103350505	olfactory receptor 52N1	-1.55
LOC100345611	olfactory receptor 4F3/4F16/4F29-like	-1.53
LOC100345265	olfactory receptor 49-like	-1.52
LOC100347240	olfactory receptor 12D3	-1.52
LOC100337750	putative olfactory receptor GPCRLTM7	-1.51
LOC100346428	olfactory receptor 8H1-like	-1.49
LOC100354590	olfactory receptor 2T11-like	-1.48
LOC100355521	olfactory receptor 7E24-like	-1.48
LOC100352387	olfactory receptor 5B12	1.48
OLFR555	olfactory receptor 555	1.48
LOC100351383	olfactory receptor 52N4-like	1.47
LOC100341839	olfactory receptor 5W2-like	-1.46
LOC100346601	olfactory receptor 5AN1-like	1.45
OLFR582	olfactory receptor 582	-1.44
LOC100357002	olfactory receptor-like protein DTMT	1.44
LOC100349877	olfactory receptor 6C74-like	-1.44
LOC100349637	olfactory receptor 52A5-like	-1.42
OLFR622	olfactory receptor 622	-1.42
LOC100345016	olfactory receptor 6C74-like	-1.41
LOC100344793	olfactory receptor 52K1-like	-1.41

Table S1.F. Top differentially expressed rabbit genes encoding a) known and b) new DED related genes that were not described before in the literature.

Gene	Gene Name	Gene Accession no.	-2>FC>2, p-value <0.01
PIP	prolactin-induced protein	NM_001171116	-24.52
LOC103347214	odorant-binding protein-like	XM_008253694	-18.52
LOC103347775	uncharacterized LOC103347775	XM_008254700	9.74
HBB2	hemoglobin, beta	NM_001082260	8.49
S100A12	S100 calcium binding protein A12	XM_002715516	8.40
SCGB2A1	lipophilin CP	NM_001082096	-7.40
ORM1	orosomucoid 1	NM_001101695	6.91
SCGB1D1	secretoglobin family 1D member 2-like	XM_002721047	-6.90
SCGB1D	lipophilin BL	NM_001082157	-6.66
CYP2A10	cytochrome P450 2A10	NM_001171049	-6.26
ENSOCUG00000026544	biotype:protein_coding	ENSOCUT00000025645	-6.16
ALAS2	aminolevulinate, delta-, synthase 2	XM_002720030	6.16
HBA	Oryctolagus cuniculus alpha-hemoglobin (HBA), mRNA.	NM_001082389	5.92
LOC100355142	transmembrane protease serine 11B-like	XM_008267890	5.43
LOC103347507	major allergen I polypeptide chain 1-like	XM_008254014	-5.22
LOC100008793	lipophilin AS	ENSOCUT00000000455	-5.00
LOC100349251	UPF0762 protein C6orf58-like	XM_002714721	-4.88
ENSOCUG00000025374	ensembl:known chromosome:OryCun2.0:2:98921851:98926926:1 gene:ENSOCUG00000025374 gene_biotype:protein_coding transcript_biotype:protein_coding	ENSOCUT00000030730	4.71
VNN3	vascular non-inflammatory molecule 3	XM_002714826	-4.69
LOC100350165	cysteine-rich secretory protein 3-like	XM_002714381	4.28
C13H1orf162	chromosome 13 open reading frame, human C1orf162	XM_008264672	4.09
IDO1	indoleamine 2,3-dioxygenase 1	XM_002720800	4.08
CHI3L1	chitinase 3-like 1 (cartilage glycoprotein-39)	XM_008268558	3.89
IFGGA1	interferon-inducible GTPase 1	XM_002710316	3.79
SCGB1D1	lipophilin AL2	XM_008274326	-3.78
S100A8	S100 calcium binding protein A8	XM_002715343	3.72
LYPD1	LY6/PLAUR domain containing 1	XM_002712411	-3.69
CXCL6	alveolar macrophage chemotactic factor-like	XM_002717113	3.54
LOC100341513	uncharacterized LOC100341513	XR_516721	-3.47
LOC100347536	NXPE family member 3-like	XM_002721869	-3.47
ENSOCUG00000024628	ensembl:known scaffold:OryCun2.0:AAGW02080938:949:18810:1 gene:ENSOCUG00000024628 gene_biotype:protein_coding transcript_biotype:protein_coding	ENSOCUT00000000994	3.45
IRG1	immunoresponsive 1 homolog (mouse)	XM_002712932	3.43
DCT	dopachrome tautomerase	NM_001297493	-3.37
PRG4	proteoglycan 4	XM_008268756	3.36

GYPA	glycophorin A (MNS blood group)	XM_008267405	3.28
LOC100349428	PREDICTED: Oryctolagus cuniculus cytochrome c oxidase protein 20 homolog (LOC100349428), transcript variant X2, mRNA.	XM_002717638	-3.26
HRH4	histamine receptor H4	XM_002713460	3.23
HLA-DQA1	SLA class II histocompatibility antigen, DQ haplotype D alpha chain-like	XM_002714510	3.17
SAA3	serum amyloid A-3	NM_001082302	3.17
IRGM	immunity-related GTPase family, M	XM_002710166	3.15
ENSOCUG00000026373	ensembl:known scaffold:OryCun2.0:AAGW02083105:366:1905:-1 gene:ENSOCUG00000026373 gene_biotype:protein_coding transcript_biotype:protein_coding	ENSOCUT00000031889	-3.09
KIAA1324	KIAA1324 ortholog	XM_008264757	-3.07
CXCL11	chemokine (C-X-C motif) ligand 11	XM_002717006	3.07
MMP12	matrix metallopeptidase 12 (macrophage elastase)	NM_001082771	3.06
LOC103347413	uncharacterized LOC103347413	XR_516148	2.95
MPEG1	macrophage expressed 1	XM_002709176	2.94
mainchrUn_0038+11746781194498	0	0	-2.94
LOC100354078	cytochrome P450 2F5-like	XM_002722297	-2.93
MUC12	mucin-12-like	XM_008253995	2.87
HLA-DMB	histocompatibility antigen DM heterodimer light chain-like	NM_001190433	2.86
COCH	cochlin	XM_002718124	-2.86
LOC100353150	glycine N-phenylacetyltransferase-like	XM_002709174	2.82
ENSOCUG00000028063	ensembl:known scaffold:OryCun2.0:AAGW02082581:1016:2386:1 gene:ENSOCUG00000028063 gene_biotype:protein_coding transcript_biotype:protein_coding	ENSOCUT00000031956	-2.80
CYP3A6	cytochrome P450 3A6	NM_001171268	2.79
ENSOCUG00000024597	ncrna:novel chromosome:OryCun2.0:15:63037739:63037867:1 gene:ENSOCUG00000024597 gene_biotype:snoRNA transcript_biotype:snoRNA	ENSOCUT00000026471	-2.78
ABCA6	ATP-binding cassette, sub-family A (ABC1), member 6	XM_008271750	2.77
CA1	carbonic anhydrase I	XM_008255708	2.77
LTF	lactotransferrin	XM_008260521	-2.76
ENSOCUG00000027090	ensembl:known chromosome:OryCun2.0:21:4608079:4608673:1 gene:ENSOCUG00000027090 gene_biotype:protein_coding transcript_biotype:protein_coding	ENSOCUT00000024792	2.75
ENSOCUG00000024209	ncrna:novel scaffold:OryCun2.0:GL018730:1477043:1477139:-1 gene:ENSOCUG00000024209 gene_biotype:rRNA transcript_biotype:rRNA	ENSOCUT00000025885	2.74
ENSOCUG00000025220	ensembl:known chromosome:OryCun2.0:21:4632524:4633098:1 gene:ENSOCUG00000025220 gene_biotype:protein_coding transcript_biotype:protein_coding	ENSOCUT00000032152	2.72
TYRP1	tyrosinase-related protein 1	NM_001297495	-2.69
SNCA	synuclein, alpha (non A4 component of amyloid precursor)	XM_002717000	2.68
SCGB2A1	lipophilin CL	NM_001082094	-2.68
CXCR1	chemokine (C-X-C motif) receptor 1	NM_001171082	2.67

ENSOCUG00000018736	ncrna:novel chromosome:OryCun2.0:4:10776345:10776430:1 gene:ENSOCUG00000018736 gene_biotype:miRNA transcript_biotype:miRNA	ENSOCUT00000018736	-2.60
ENSOCUG00000025019	ensembl:known chromosome:OryCun2.0:2:98814687:98815160:-1 gene:ENSOCUG00000025019 gene_biotype:protein_coding transcript_biotype:protein_coding	ENSOCUT00000006213	2.59
FGFBP1	fibroblast growth factor-binding protein 1-like	ENSOCUT00000022661	2.55
LOC100353536	protein FAM26F-like	XM_002714734	2.51
LOC100338508	UDP-glucuronosyltransferase 2B17-like	XM_002717129	2.50
CDK1	cyclin-dependent kinase 1	XM_002718473	2.48
CUBN	cubilin (intrinsic factor-cobalamin receptor)	XM_008268302	2.47
UGT2A1	UDP glucuronosyltransferase 2 family, polypeptide A1, complex locus	XM_002717124	2.46
KRT16	keratin 16	XM_002719157	2.44
KRT18	keratin 18	XM_008256498	-2.42
LOC100347041	leukocyte immunoglobulin-like receptor subfamily A member 6	XM_008253753	2.42
RPL36AL	ribosomal protein L36a-like	XM_002718228	2.39
HLA-DRB1	HLA class II histocompatibility antigen, DRB1-4 beta chain	XM_002714606	2.38
CXCL8	chemokine (C-X-C motif) ligand 8	NM_001082293	2.38
ENSOCUG00000025247	ncrna:novel chromosome:OryCun2.0:3:44630840:44630968:1 gene:ENSOCUG00000025247 gene_biotype:snoRNA transcript_biotype:snoRNA	ENSOCUT00000027529	2.38
LOC100347786	NXPE family member 3-like	XM_008248890	-2.38
CCL7	C-C motif chemokine 7-like	XM_008271107	2.37
HTR1B	5-hydroxytryptamine (serotonin) receptor 1B, G protein-coupled	NM_001082790	-2.36
ENSOCUG00000025864	ensembl:known scaffold:OryCun2.0:GL019480:44303:45623:-1 gene:ENSOCUG00000025864 gene_biotype:protein_coding transcript_biotype:protein_coding	ENSOCUT00000031158	-2.34
ENSOCUG00000027123	ncrna:novel chromosome:OryCun2.0:3:35482393:35482515:1 gene:ENSOCUG00000027123 gene_biotype:snRNA transcript_biotype:snRNA	ENSOCUT00000030529	2.33
LOC100358539	interferon-induced guanylate-binding protein 1-like	XM_002715873	2.32
LOC103347245	transmembrane protein 254-like	XM_008253729	-2.31
CYBB	cytochrome b-245 beta polypeptide	NM_001082100	2.31
SLC13A1	solute carrier family 13 (sodium/sulfate symporter), member 1	XM_008258262	2.30
NOX1	NADPH oxidase 1	XM_002720381	2.30
HLA-DRA	major histocompatibility complex, class II, DR alpha	NM_001171118	2.29
CXCR2	chemokine (C-X-C motif) receptor 2	NM_001171090	2.28
SULT6B2P	sulfotransferase 6B1-like	XM_008259553	2.28
CKAP2	cytoskeleton associated protein 2	XM_002720757	2.28
LOC100353927	glutathione S-transferase alpha I-like	XM_002714388	2.27
ENSOCUG00000023732	ncrna:novel chromosome:OryCun2.0:12:43218510:43218616:-1 gene:ENSOCUG00000023732 gene_biotype:snRNA transcript_biotype:snRNA	ENSOCUT00000025160	-2.27

ENSOCUG00000026469	ensembl:known scaffold:OryCun2.0:GL018893:510765:511058:-1 gene:ENSOCUG00000026469 gene_biotype:protein_coding transcript_biotype:protein_coding	ENSOCUT00000022049	2.27
TCN1	transcobalamin I (vitamin B12 binding protein, R binder family)	XM_002709185	-2.26
GSTA2	glutathione S-transferase Yc	NM_001171098	2.26
LOC100343992	alcohol dehydrogenase 6-like	XM_002716960	2.26
TMEM126B	transmembrane protein 126B	XM_002708660	2.25
GPR18	G protein-coupled receptor 18	XM_008260138	2.25
ENSOCUG00000024209	ncrna:novel scaffold:OryCun2.0:GL018730:1477043:1477139:-1 gene:ENSOCUG00000024209 gene_biotype:rRNA transcript_biotype:rRNA	ENSOCUT00000025885	2.25
ITGAM	integrin, alpha M (complement component 3 receptor 3 subunit)	XM_002721749	2.25
LOC100357464	UDP-glucuronosyltransferase 2B14-like	XM_008253410	-2.25
LOC100339150	serpin B4-like	XM_002713633	2.24
CD14	CD14 molecule	NM_001082195	2.23
GENSCAN00000038921	cdna:genscan scaffold:OryCun2.0:GL018893:444351:476733:-1 transcript_biotype:protein_coding	GENSCAN00000038921	2.23
ENSOCUG00000023558	ensembl:known scaffold:OryCun2.0:AAGW02081222:6661:7103:1 gene:ENSOCUG00000023558 gene_biotype:protein_coding transcript_biotype:protein_coding	ENSOCUT00000027168	2.23
LOC100357801	interferon-induced very large GTPase 1-like	XM_002708773	2.22
ENSOCUG00000020338	ncrna:novel chromosome:OryCun2.0:18:66312045:66312171:-1 gene:ENSOCUG00000020338 gene_biotype:snoRNA transcript_biotype:snoRNA	ENSOCUT00000020338	2.22
CLEC4D	C-type lectin domain family 4, member D	XM_008259781	2.22
ENSOCUG00000019855	ncrna:novel chromosome:OryCun2.0:1:123186091:123186217:1 gene:ENSOCUG00000019855 gene_biotype:snoRNA transcript_biotype:snoRNA	ENSOCUT00000019855	2.21
ENSOCUG00000003355	ensembl:known_by_projection chromosome:OryCun2.0:15:79730470:79751621:-1 gene:ENSOCUG00000003355 gene_biotype:protein_coding transcript_biotype:protein_coding	ENSOCUT00000003354	-2.21
CXCR4	chemokine (C-X-C motif) receptor 4	XM_002712124	2.21
ERICH4	glutamate-rich 4	XM_002722292	2.21
GENSCAN00000028894	cdna:genscan scaffold:OryCun2.0:GL019212:88257:106671:1 transcript_biotype:protein_coding	GENSCAN00000028894	2.21
FRZB	frizzled-related protein	XM_002712232	-2.19
ENSOCUG00000025999	ncrna:novel chromosome:OryCun2.0:11:84957233:84957341:1 gene:ENSOCUG00000025999 gene_biotype:snRNA transcript_biotype:snRNA	ENSOCUT00000028729	2.18
ENSOCUG00000013533	ensembl:known_by_projection scaffold:OryCun2.0:GL019133:69902:74082:-1 gene:ENSOCUG00000013533 gene_biotype:protein_coding transcript_biotype:protein_coding	ENSOCUT00000013531	2.18
LOC100351163	HLA class II histocompatibility antigen, DQ beta 1 chain	XM_008262757	2.17
SLC7A3	solute carrier family 7 (cationic amino acid transporter, y+ system), member 3	XM_008272757	-2.17
PPBP	pro-platelet basic protein (chemokine (C-X-C motif) ligand 7)	XM_008267883	2.16

CLEC7A	C-type lectin domain family 7, member A	XM_002712883	2.15
CYP2B4	cytochrome P450, family 2, subfamily b, polypeptide 4	ENSOCUT00000023979	-2.15
LOC100357801	interferon-induced very large GTPase 1-like	XM_002708773	2.14
SLC12A1	solute carrier family 12 (sodium/potassium/chloride transporter), member 1	NM_001170971	2.14
LOC100348219	solute carrier family 7 member 13-like	XM_002710566	2.14
ACMSD	aminocarboxymuconate semialdehyde decarboxylase	XM_008258506	2.14
AZGP1	alpha-2-glycoprotein 1, zinc-binding	XM_002721874	2.12
PLA2G7	phospholipase A2, group VII (platelet-activating factor acetylhydrolase, plasma)	XM_008263011	2.11
PTPRC	protein tyrosine phosphatase, receptor type, C	XM_008268693	2.11
CCNB2	cyclin B2	XM_002718205	2.11
CD74	CD74 molecule, major histocompatibility complex, class II invariant chain	XM_008255262	2.11
LOC100352971	aldo-keto reductase family 1 member C1 homolog	XM_008248597	2.11
LOC100353656	uncharacterized LOC100353656	XM_008253672	2.11
CTHRC1	collagen triple helix repeat containing 1	XM_002710591	2.09
PANX2	lipophilin AL	NM_001082092	-2.09
EVI2B	ecotropic viral integration site 2B	XM_002718938	2.08
MKI67	marker of proliferation Ki-67	XM_008274847	2.08
CXCL10	chemokine (C-X-C motif) ligand 10	XM_002717106	2.07
ENSOCUG00000019045	ncrna:novel chromosome:OryCun2.0:12:123077079:123077210:1 gene:ENSOCUG00000019045 gene_biotype:snoRNA transcript_biotype:snoRNA	ENSOCUT00000019045	-2.06
LGI1	leucine-rich, glioma inactivated 1	XM_002718517	-2.06
ENSOCUG00000020448	ncrna:novel chromosome:OryCun2.0:2:32523488:32523588:-1 gene:ENSOCUG00000020448 gene_biotype:snRNA transcript_biotype:snRNA	ENSOCUT00000020448	-2.06
ACSM3	acyl-CoA synthetase medium-chain family member 3	XM_002711809	2.06
STC1	stanniocalcin 1	XM_002709464	-2.05
TFEC	transcription factor EC	XM_002712010	2.05
BPGM	2,3-bisphosphoglycerate mutase	NM_001082269	2.04
LRP2	low density lipoprotein receptor-related protein 2	XM_008258707	2.04
SLC5A12	solute carrier family 5 (sodium/monocarboxylate cotransporter), member 12	XM_002709020	2.03
LOC100348998	UPF0762 protein C6orf58-like	XM_002714720	-2.03
SPRR3	small proline-rich protein 3	XM_002715530	-2.03
ENSOCUG00000028250	ncrna:novel chromosome:OryCun2.0:3:28831007:28831116:-1 gene:ENSOCUG00000028250 gene_biotype:rRNA transcript_biotype:rRNA	ENSOCUT00000032245	-2.03
LCN2	lipocalin 2	XM_002722973	-2.03
ENSOCUG00000022278	ensembl:known scaffold:OryCun2.0:AAGW02080074:40327:40629:1 gene:ENSOCUG00000022278 gene_biotype:protein_coding transcript_biotype:protein_coding	ENSOCUT00000030189	2.03
LOC100339545	olfactory receptor 2G6-like	XM_008253543	-2.03
LOC100345057	zymogen granule membrane protein 16-like	XM_002724277	2.03
LRRC19	leucine rich repeat containing 19	XM_002708045	2.02

ENSOCUG00000027540	ncrna:novel chromosome:OryCun2.0:3:81502617:81502733:-1 gene:ENSOCUG00000027540 gene_biotype:snRNA transcript_biotype:snRNA	ENSOCUT00000031141	2.02
ENSOCUG00000023169	ensembl:known scaffold:OryCun2.0:GL019400:39024:39463:-1 gene:ENSOCUG00000023169 gene_biotype:protein_coding transcript_biotype:protein_coding	ENSOCUT00000023469	2.02
LOC100357038	UDP-glucuronosyltransferase 2B31-like	XM_002724185	2.02
LOC100340280	putative P2Y purinoceptor 10	XM_008272964	2.02
TMEM27	transmembrane protein 27	XM_002719875	2.02
ENSOCUG00000021365	ensembl:known chromosome:OryCun2.0:10:21525526:21525991:-1 gene:ENSOCUG00000021365 gene_biotype:protein_coding transcript_biotype:protein_coding	ENSOCUT00000022628	2.01
SLC17A1	solute carrier family 17 (organic anion transporter), member 1	NM_001082307	2.01
ENSOCUG00000024554	ncrna:novel chromosome:OryCun2.0:17:13378531:13378693:-1 gene:ENSOCUG00000024554 gene_biotype:snRNA transcript_biotype:snRNA	ENSOCUT00000026401	2.01
NR1D1	nuclear receptor subfamily 1, group D, member 1	XM_002719349	-2.01
ENSOCUG00000023388	ncrna:novel scaffold:OryCun2.0:GL018717:1518910:1519100:1 gene:ENSOCUG00000023388 gene_biotype:snRNA transcript_biotype:snRNA	ENSOCUT00000024628	2.01
ENSOCUG00000027378	ensembl:known_by_projection scaffold:OryCun2.0:GL018878:166737:168016:1 gene:ENSOCUG00000027378 gene_biotype:protein_coding transcript_biotype:protein_coding	ENSOCUT00000025993	2.01
SLAMF6	SLAM family member 6	XM_008264216	2.00
FABP1	fatty acid binding protein 1, liver	XM_002709637	2.00
ENSOCUG00000021204	ncrna:novel chromosome:OryCun2.0:4:1948856:1948986:1 gene:ENSOCUG00000021204 gene_biotype:snoRNA transcript_biotype:snoRNA	ENSOCUT00000021346	2.00
WIF1	WNT inhibitory factor 1	XM_008256742	-2.00
ENSOCUG00000019629	ncrna:novel scaffold:OryCun2.0:GL018744:1656831:1656963:-1 gene:ENSOCUG00000019629 gene_biotype:snoRNA transcript_biotype:snoRNA	ENSOCUT00000019629	2.00
ENSOCUG00000027549	ensembl:known scaffold:OryCun2.0:GL019873:1:11514:-1 gene:ENSOCUG00000027549 gene_biotype:protein_coding transcript_biotype:protein_coding	ENSOCUT00000021541	-2.00

Rabbit Con A Model, samples collected in 18th day of the experiment.

Genes listed above were identify using Affymetrix GeneChip™ Rabbit Gene 1.0 ST Array (ThermoFisher Scientific) containing 496,321 probes to assess 23,364 transcripts, transcript variants and alternative splicing events. Each transcript was measured with a median of 22 probes. Of these 23,364 transcripts, 14,713 (63%) are fully annotated, with known homology to human genes (HomoGene NCBI database) while 8,651 (37%) are partially annotated without clear homology to human genes.
